# Successful field performance in warm and dry environments of soybean expressing the sunflower transcription factor HB4

**DOI:** 10.1093/jxb/eraa064

**Published:** 2020-03-06

**Authors:** Karina F Ribichich, Mariana Chiozza, Selva Ávalos-Britez, Julieta V Cabello, Augustin L Arce, Geronimo Watson, Claudia Arias, Margarita Portapila, Federico Trucco, Maria E Otegui, Raquel L Chan

**Affiliations:** 1 Instituto de Agrobiotecnología del Litoral, Universidad Nacional del Litoral – CONICET, Facultad de Bioquímica y Ciencias Biológicas, Santa Fe, Argentina; 2 INDEAR/BIOCERES, Rosario, Argentina; 3 Estación Experimental Pergamino, Instituto Nacional de Tecnología Agropecuaria (INTA), Pergamino, Argentina; 4 CIFASIS, Universidad Nacional de Rosario – CONICET, Rosario, Argentina; 5 CONICET-INTA-FAUBA, Estación Experimental Pergamino, Facultad de Agronomía Universidad de Buenos Aires, Buenos Aires, Argentina; 6 University of Essex, UK

**Keywords:** Drought tolerance, HaHB4, photosynthesis rate, seed yield determination, soybean field trials, sunflower transcription factor, transgenic soybean, water use efficiency

## Abstract

Soybean yield is limited primarily by abiotic constraints. No transgenic soybean with improved abiotic stress tolerance is commercially available. We transformed soybean plants with genetic constructs able to express the sunflower transcription factor HaHB4, which confers drought tolerance to Arabidopsis and wheat. One line (b10H) carrying the sunflower promoter was chosen among three independent lines because it exhibited the best performance in seed yield, and was evaluated in the greenhouse and in 27 field trials in different environments in Argentina. In greenhouse experiments, transgenic plants showed increased seed yield under stress conditions together with greater epicotyl diameter, larger xylem area, and increased water use efficiency compared with controls. They also exhibited enhanced seed yield in warm and dry field conditions. This response was accompanied by an increase in seed number that was not compensated by a decrease in individual seed weight. Transcriptome analysis of plants from a field trial with maximum difference in seed yield between genotypes indicated the induction of genes encoding redox and heat shock proteins in b10H. Collectively, our results indicate that soybeans transformed with *HaHB4* are expected to have a reduced seed yield penalty when cultivated in warm and dry conditions, which constitute the best target environments for this technology.

## Teaser

HaHB4 transgenic soybean plants significantly outyielded controls in warm and dry environments, showing increased seed number, xylem area, and water use efficiency, and induction of genes encoding redox and heat shock proteins.

## Introduction

Soybean (*Glycine max* L. Merr.) is one of the most important crops worldwide and has a wide range of uses. Many countries have adopted biotech soybeans, which currently cover more than 90% of the land cropped to this species in the USA and Argentina (two of the four main producers) (http://www.isaaa.org/). However, biotic and abiotic constraints still limit the seed yield (SY) and seed quality of this species (Hartman *et al.*, 2011).

Approved and commercialized genetically modified (GM) soybeans show resistance to herbicide or herbivore attack, but to date there are no GM soybeans with increased abiotic stress tolerance available on the worldwide market. Among other species, increased seed yield (SY) of GM drought-tolerant maize (*Zea mays* L.) grown under water-limited conditions was described by Castiglioni *et al.* (2008). These maize plants express the bacterial RNA chaperones CspB and CspA, which generate tolerance to drought as well as to cold and heat (Liang, 2017).

With regard to transgenic (TG) soybean, besides the work devoted to glyphosate technology, scientific literature considering other traits is scarce, and almost absent in terms of field trials. A few publications have reported evaluations of TG soybean, mostly in greenhouse or growth chamber conditions. One such is example is that of soybean plants overexpressing *GmSYP24*, a dehydration-responsive gene, which showed insensitivity to osmotic/drought and high salinity stresses via stomatal closure involving an abscisic acid (ABA) signal pathway in experiments carried out in the greenhouse (Chen *et al.*, 2019). Another work described the overexpression of the basic leucine-zipper transcription factor (TF) GmFDL19, resulting in early flowering and enhanced tolerance to drought and salt stress; however, the SY in stress or standard growth conditions was not reported (Li *et al.*, 2017). In another study, a soybean MYB encoding gene was overexpressed in soybean plants. TG soybean plants carrying an extra copy of *GmMYB8*4 (a R2R3-MYB TF) exhibited a higher survival rate after severe drought when they were tested in controlled conditions in a growth chamber (Wang *et al.*, 2017).

Water deficit is the most important factor affecting crop SY worldwide. Water scarcity for agriculture increases production costs and indicates the need to improve water resource use efficiency across a broad range of permanent and transient drought-prone regions of the world. Drought tolerance without yield penalties is a desirable trait, but is difficult to achieve. Plants that have evolved to survive under water deficit conditions display physiological changes, including in particular stomatal closure. Most of the genes that are positively involved in the drought response and which have been tested both in model plants and crops induce stomatal closure and, hence, increase plant survival, but reduce biomass and seed production under the mild stress conditions that are very frequently encountered in field conditions (Skirycz *et al.*, 2011). Moreover, Passioura (2012) analyzed a huge number of reports referring to drought-tolerant *Arabidopsis thaliana* (L.) Heynh. plants and identified that the enhanced survival of a high percentage of these plants was simply explained by their reduced size, and concomitant slower water uptake, relative to wild-type (WT) plants (Morran *et al.*, 2011).

TFs are particularly abundant in the plant kingdom, representing ~6% of encoded proteins (Riechmann, 2002). Among plant TFs, the homeodomain-leucine zipper (HD-Zip) proteins are unique to plants and have been assigned roles in development associated with environmental stress factors (Ariel *et al.*, 2007; Perotti *et al.*, 2017). Although they have conserved structures and functions, HD-Zip I TFs diverged during evolution, and show different features among different plant species. Soybean plants have 36 members of the HD-Zip I family (Belamkar *et al.*, 2014). Among them, *GmHB6*, *GmHB13*, and *GmHB21* showed different expression levels after drought treatment in susceptible (BR 16) and tolerant (EMBRAPA 48) soybean cultivars, indicating the presence of different regulatory *cis*-acting elements. In particular, *GmHB13* was exclusively induced by water deficit in the drought-tolerant cultivar, whereas *GmHB6* was repressed only in the susceptible cultivar (Pereira *et al.*, 2011). Functional studies of these TFs are not available in the scientific literature to date, but their differential expression in tolerant and susceptible cultivars suggests that they have a role in the response to drought.

Sunflower (*Helianthus annuus* L.) belongs to the Asteraceae clade of the angiosperms and has several divergent HD-Zip I members (Arce *et al.*, 2011). Among these, HaHB4 (*Helianthus annuus* HomeoBox 4) has been well characterized. This TF exhibits an abnormally short carboxy-terminus compared with the HD-Zip I members in Arabidopsis, and its expression is highly induced by various environmental factors (drought, salinity, darkness) and plant hormones (ethylene, ABA, jasmonic acid) (Gago *et al.*, 2002; Manavella *et al.*, 2006, 2008a, *b*, *c*). Arabidopsis plants expressing this sunflower TF either under a constitutive or an inducible promoter (Cabello *et al.*, 2007) exhibited enhanced tolerance to water deficit. Recently, it was reported that HaHB4 was able to confer drought tolerance to wheat (*Triticum aestivum* L.) plants tested in greenhouses and in 37 field trials (González *et al.*, 2019). It was proposed as part of a potential molecular mechanism that this TF could interact with endogenous members of the same family by dimerization or by protein–DNA interactions (Gonzalez *et al.*, 2019).

In this work we show that HaHB4 was able to confer drought tolerance and increased SY to soybean plants tested in the field, particularly in warm and dry environments. Moreover, we show that the improved performance of HaHB4 plants is strongly related to enhanced water use, biomass production, and water use efficiency (WUE), as well as to changes in plant architecture. Transcriptome analyses performed with field-harvested samples indicated that genes encoding redox and heat shock proteins are induced in the TG genotype. This work constitutes a multidisciplinary approach that contributes to the understanding of the abiotic stress tolerance mechanisms displayed in soybean by the introduction of the sunflower TF HaHB4.

## Materials and methods

### Genetic constructs

The open reading frame of cDNA encoding full-length *HaHB4* cloned into the *Bam*HI/*Sac*I sites of pBluescript SK- (Stratagene, Uppsala, Sweden) was used as template in a PCR with oligonucleotides H4-F (5′-ATGTCTCTTCAACAAGTAACAACCACCAGG-3′) and Transf2 (5′-GCCGAGCTCTTAGAACTCCCACCACTTTTG-3′), which included initiation and stop codons. The PCR amplification product was cloned into a pGEM®-T-Easy vector (Promega, Madison, WI, USA) and named pHaHB4.2. Then, the cDNA was cloned into expression cassettes bearing two different promoters: (i) the constitutive *35S CaMV* promoter (*35S:HaHB4.2*) and (ii) the inducible *HaHB4* promoter. Both cassettes were subcloned into a vector carrying the *bar* gene and the NOS terminator sequence (Chan and Gonzalez, 2013). Clones were obtained in *Escherichia coli* and then *Agrobacterium tumefaciens* (strain EHA101) was transformed. The sequences were checked (Macrogen, Korea) and, as previously described, a few mutations were detected (Chan and Gonzalez, 2013; González *et al.*, 2019). Transactivation activity and other characteristics of these point mutants were described in detail in González *et al.* (2019).

### Plant transformation and selection of transgenic events

Soybean TG events were generated using an *Agrobacterium*-mediated protocol (Somers *et al.*, 2003) and the cultivar Williams 82 (W82) according to the methods described by Höfgen and Willmitzer (1988). TG events were selected using ammonium glufosinate. T_1_ seeds were obtained for 35 independent events.

Multiplication of the transformed cells was conducted in a greenhouse. T_1_ individuals derived from each event were sampled for a segregation test by PCR determination. Lines derived from selfings of individuals from selected events (3:1 segregation in T_1_) were sown and analyzed by PCR to identify homozygous lines, as indicated by the absence of negative segregants among the sampled progeny (at least five individuals were sampled per line). Seed augmentation (T_3_ seed) of single-copy homozygous events was conducted in a greenhouse.

### Field trials for event selection and testing of cultivars

The experimental network for the evaluation of HaHB4 effects in soybeans included 30 field experiments conducted across a wide range of environments through six growing seasons and at 16 sites. Experiments were organized into three groups. Group 1 corresponded to the evaluation of three TG events (a11H, a5H, and b10H) in comparison with the WT parental cultivar W82, and included 17 experiments conducted during 2009–2010 and 2012–2013. Some of these experiments were also included as part of Group 2 (described below). General growing conditions (i.e. cumulative incident solar radiation, mean temperatures, rainfall, and potential evapotranspiration) during the cycle experienced by crops in 14 of these experiments are described in Supplementary Table S1 at *JXB* online. Group 2 corresponded to the analysis of the best-performing TG event (b10H) in comparison with the WT parental cultivar W82 for the detection of genotype by environment (G×E) interactions, and included 27 experiments carried out during 2009–2010 and 2018–2019. For this group, an environmental index (EI) was computed as the average SY or SY component (seed numbers, individual seed weight) of all evaluated genotypes in a given environment. Each trait of b10H and of W82 in each environment was regressed with respect to the corresponding EI. Growing conditions of all experiments in Group 2 are described in Supplementary Table S1. Rainfall data were obtained *in situ* and other weather records were obtained from the nearest weather station (http://siga2.inta.gov.ar). Water balance for different growth periods and for the whole cycle was obtained as the difference between potential evapotranspiration (PET, in mm) and water supplied by rainfall (Rain, in mm) plus irrigation (IR, in mm). The relative water balance (RWB) was computed as in equation 1:

$$\begin{array}{l} RWB=\frac{Rain+IR-PET}{PET} \end{array}$$ (1)

Group 3 focused on the detection of differences in the physiological determination of SY between b10H and W82, which was performed in 4 of the 27 experiments in Group 2 (those carried out during 2017–2018 and 2018–2019).

Some experiments were carried out in the same year × site combination but using different sowing dates (e.g. the Aranguren-2013, Carmen de Areco, Roldán, and San Agustín sites), water regimes (e.g. Liborio Luna and Pergamino), or rates of phosphorus fertilizer application (e.g. Aranguren-2014). The SY of all cultivars was always assessed in a randomized complete block design with at least three replicates and plots of at least 10.4 m^2^ (2.08 m width × 5 m length). Plots were machine-sown in all experiments except those performed at Pergamino and IAL-Santa Fe (2017–2018), which were hand-sown. Sowing took place between 7 November and 14 January and harvest occurred between 28 March and 9 May, and the stand density ranged between 30 and 40 plants m^−2^.

In the 2017–2018 water-deficit experiment conducted at Pergamino, rainfall was excluded from plots by means of removable shelters installed 23 d after sowing (i.e. before the start of stage R1 of development on 39 d after sowing; Fehr and Caviness, 1977) and removed 91 d after sowing (~R6). In this experiment, soil water content was surveyed from 23 d after sowing up to R7 (on 110 d after sowing) by means of volumetric measurements (0–30 cm depth) and neutron probe (Troxler 3400, Troxler Electronic Laboratories, NC, USA) measurements (30–185 cm depth). The difference between successive soil water measurements plus the amount of Rain+IR water added to each plot allowed estimation of crop water use (crop evapotranspiration; ET_C_, in mm) during this period. All experiments were kept free of weeds, insects, and diseases by means of the necessary recommended controls.

### Crop and plant phenotyping in field experiments

Days to stages R1 and R7 were assessed in 11 experiments of Group 1 for the analysis of event effects on phenology. In all experiments, SY was obtained at R8 by machine (all experiments except those performed at the Pergamino and IAL-Santa Fe sites) or hand harvesting (at the Pergamino and IAL-Santa Fe sites) of all plants present in at least 1 m^2^ of a central row of each plot, which were threshed for seed recovery. Seeds were cleaned and weighed, and seed weight was corrected for estimation of SY (in g m^−2^) on a 13% moisture basis. The relative SY of each experiment was computed by using equation 2:

$$\begin{array}{l} RSY=\frac{SY_{TG}-SY_{WT}}{SY_{WT}} \end{array}$$ (2)

where SY_TG_ and SY_WT_ represent the SY of the TG event b10H and of the WT parental cultivar W82, respectively.

The number of seeds and individual seed weight were assessed in 17 experiments of Group 1, 23 experiments of Group 2, and all experiments of Group 3. For this purpose, at least three samples of 100 seeds each were taken from the seed bulk and weighed; the obtained values were averaged for estimation of individual seed weight (in mg). Seed number was computed as the ratio of SY to individual seed weight and expressed on a per m^2^ basis.

Total aerial crop biomass per m^2^ (BIOM m^−2^, in g m^−2^), pod biomass per m^2^ (POD_B_ m^–2^, in g m^−2^), and pod numbers per m^2^ (POD_N_ m^−2^) were surveyed at physiological maturity (R7) at the Pergamino and IAL-Santa Fe sites. For this purpose, plants present in a 0.52 m^2^ area were collected from a central row in each plot and dried at 60 ºC until constant weight was reached. The number of pods with at least one developed seed was counted on these plants. Biomass partitioning to reproductive organs at R7 was estimated as (i) the ratio of POD_B_ m^−2^ to BIOM m^−2^, described as biomass partitioning index to pods (BPI_P_), and (ii) the ratio of SY to BIOM m^−2^, described as harvest index. At Pergamino, WUE based on ET_C_ was computed for biomass (WUE_B,ETc_) and seed (WUE_SY,ETc_) production. The former was calculated as the ratio of BIOM m^−2^ to ET_C_, and the latter was calculated as the ratio of SY to ET_C_.

At the IAL site, plants at reproductive stages R1, R3, and R5 were evaluated, including measurement of light interception with a ceptometer (Cavadevices, Argentina) as described by Maddoni and Otegui (1996). Midpoint internode diameters (hypocotyls and epicotyls) were measured on three plants at stage V2, and branches per plant were measured on three plants per plot at R5. Stem sections were collected from the same region and treated as described below. Relative xylem area was estimated as the ratio of xylem area to total stem area measured with ImageJ (Rasband, 1997–2018).

### Greenhouse growth conditions and plant phenotyping

A greenhouse experiment was performed at the IAL site. Seeds of WT (W82) and TG (b10H) plants were sown and grown in 0.5 litre pots filled with white peat (Klasmann-Deilmann TS1) for 2 weeks in a culture chamber (18 h light photoperiod, 23±1 °C). Then, 14 plants per genotype were individually transferred to 8 litre pots filled with peat (Terrafertil Growmix Multipro):perlite (3:1) and 1.25 g l^−1^ of slow-release fertilizer (Compost Expert Basacote Plus) and grown until harvest in a greenhouse under temperature and humidity monitoring. One week after the plants were placed in the greenhouse, 50% of the plants were subjected to mild water stress by watering the pots to 60% of field capacity up to R3 (53 d). The rest of the plants (controls) remained well watered to 100% of field capacity during the treatment period, and pots of all plants were watered up to field capacity from R3 onwards.

Plant water use was estimated from the difference in successive weights of pots maintained at 100% and 60% of field capacity, considering the weight of plants and the soil evaporation (plants at V5 and older cover the pot surface) as negligible. Relative cumulative water use was computed for each plant as total water (ml) added during the treatment period and expressed as the ratio of water use between water-deficit and control plants of each genotype up to each evaluated time interval. Yield components, plant height, and number of branches, internodes, and pods per plant were recorded at final harvest. Midpoint internode diameters (epicotyl and first, second, and third internodes) were measured at V5 on three or four plants that had been exposed (no water added) or not exposed (irrigated) to water deficit between V3 and V5. Stem section area and xylem area were estimated with ImageJ (Rasband, 1997–2018).

### Histological and microscopic analyses

Stem sections of 0.5–1.0 cm length were collected and fixed at room temperature for 24 h in FAA solution (3.7% formaldehyde, 5% acetic acid, and 50% ethanol), and then subjected to standard alcohol series dehydration and paraffin (Histoplast; Biopack™, Argentina) embedding protocols (D’Ambrogio de Argueso, 1986). Transverse stem sections (10 µm thick) were obtained using a microtome (RM2125, Leica). Sections were mounted on slides coated with 50 mg ml^–1^ poly-d-lysine (Sigma Chemical Co., St. Louis, MO, USA) in 10 mM Tris–HCl, pH 8.0, and dried for 16 h at 37 °C. After removing the paraffin, the slices were treated with safranin/fast green stain (D’Ambrogio de Argueso, 1986), and mounted on Canadian balsam (Biopack™, Argentina) for microscopic examination in an Eclipse E200 Microscope (Nikon, Tokyo, Japan) equipped with a Nikon Coolpix L810 camera.

### Evaluation of photosynthetic parameters

Photosynthetic parameters were measured in TG (b10H) and WT (W82) soybeans during the field trial in 2017–2018 at the IAL site. Measurements were made on healthy and fully expanded leaves of randomly chosen plants at different growth stages (R3, R5, and R6). The net photosynthetic rate was assessed with a portable photosynthesis system (LI-COR, Lincoln, NE, USA). Photosynthetically active radiation (PAR) was provided by a LED light source set to 1500 μmol m^−2^ s^−1^, air flow rate through the sample chamber was set at 500 μmol^−1^ s^−1^, and CO_2_ concentration was 400 μmol mol^−1^. The air relative humidity range was 50–60% and leaf temperature range was 25–30 °C.

### Transcriptome analysis by RNA-seq

Three leaf fragments (~1 cm^2^ each) of 8–10 plants per plot were collected at the IAL field assay, placed in liquid nitrogen, and stored at –80ºC. Samples from R5–R6 were used for RNA-seq. Total RNA was extracted from pulverized samples with RNAeasy (Qiagen). RNA quality and integrity were checked by absorbance (A) measurements (A260/A280 >1.8, A260/A230 >2.0) and electrophoresis. RNA was analyzed by BGI (San Jose, USA) by sequencing eight libraries. An average of 82 468 181 clean reads/sample with more than 95% of the bases having a Phred value>20 were reported.

Raw paired-end reads were first quality trimmed with Trimmomatic (version 0.36; Bolger *et al.*, 2014) and then aligned to the *Glycine max* W82 genome, v4 (Schmutz *et al.*, 2010; from Phytozome V13, Goodstein *et al.*, 2012) using STAR (version 2.5.2b, Dobin *et al.*, 2013) with a maximum intron length of 1200 bp. Using samtools (version 1.8; Li *et al.*, 2009), only primary alignments with a minimum MAPQ of 3 were kept. Read quality before and after trimming was analyzed with FastQC (version 0.11.5; Andrews, 2010) and, together with mapping efficiency, was summarized with MultiQC (version 1.7; Ewels *et al.*, 2016). Read counts on each gene were calculated with featureCounts (version 1.6.2; Liao *et al.*, 2014) using the gene and exon annotation from Phytozome (v13; Goodstein *et al.*, 2012). Differentially expressed genes (DEGs) were determined with DESeq2 (Love *et al.*, 2014; R Core Team, 2018), filtering out genes with counts below 10 in all samples. This analysis pipeline was run with the aid of the Snakemake workflow engine (Köster and Rahmann, 2018). Gene ontology analysis was performed online with agriGO (v2; Tian *et al.*, 2017).

### Statistical analyses

Differences in SY and its components between WT cultivar W82 and TG events (experiments in Group 1), and between W82 and TG event b10H (experiments in Groups 2 and 3 as well as in the greenhouse), were assessed by ANOVA, with genotypes (G) and environments (E) as fixed factors and replicates nested within environments. A Tukey test was used for comparison of main and interaction (G×E) effects. Square root transformation was used to transform discrete variables. Other traits within a given environment were evaluated by a *t*-test. The relationship between variables was evaluated by correlation and regression analyses.

The evaluation of photosynthetic parameters was performed using the statistical software package SPSS 20.0 (SPSS Inc., Chicago, IL, USA).

### Accession numbers

For sunflower *HaHB4*, accession numbers in the EMBL, GenBank, and DDBJ Nucleotide Sequence Databases are AF339748 and AF339749. The IDs of differentially expressed soybean genes identified in the RNA-seq are listed in Supplementary Table S2.

## Results

### A set of field trials allowed the selection of a soybean transgenic *HaHB4* event

Different TG lines bearing either the constitutive 35S (lines called “a”) or the *HaHB4* (lines called “b”) promoter were obtained and, together with the WT cultivar W82, were multiplied and evaluated in field trials. After a first assessment, three independent events (a5H, a11H, and b10H) bearing only one copy of the transgene were selected for further analysis.

**Fig. 1.**
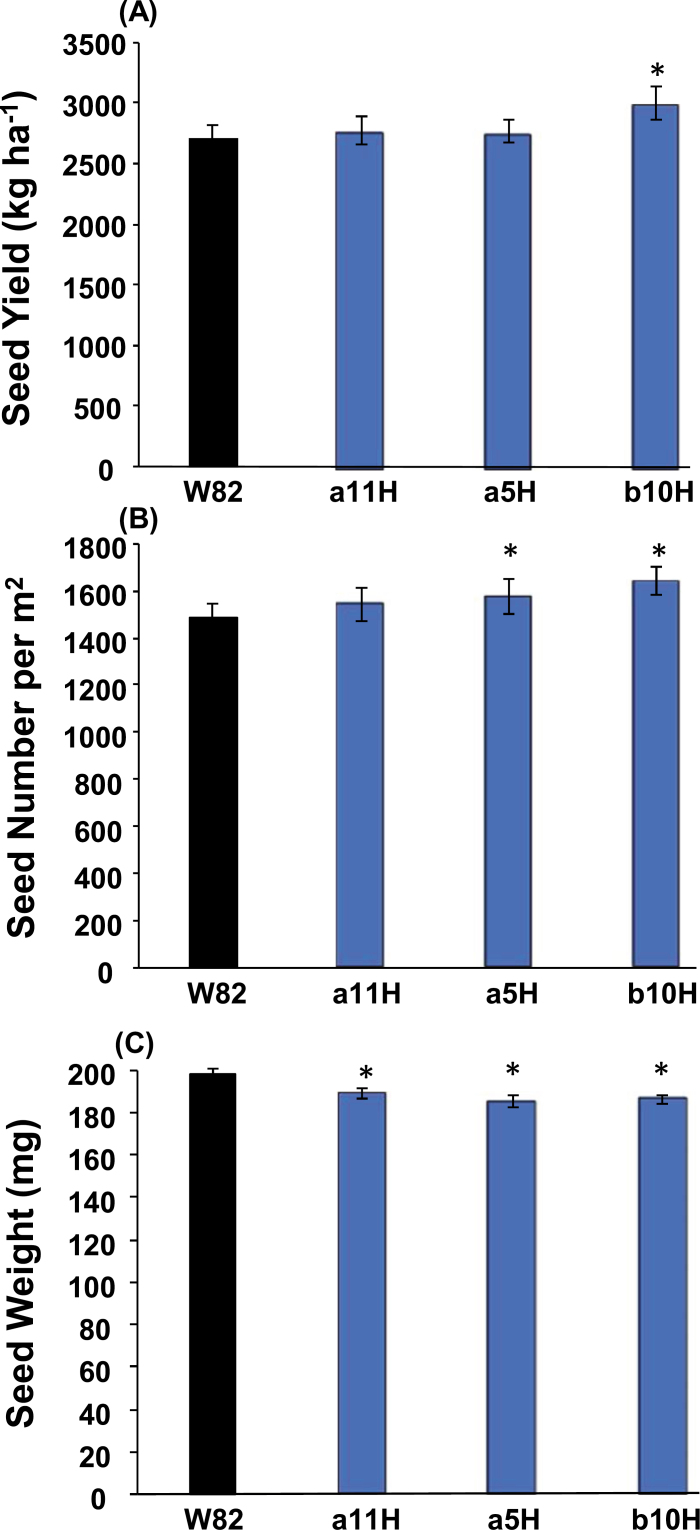
Comparison of the performance of three transgenic soybean lines and the wild-type parental cultivar W82. (A) Seed yield; (B) seed number; (C) individual seed weight. Data are mean values ±SEM×2 for the four soybean genotypes. Asterisks indicate significant differences (*P*<0.01) from the wild type.

From the experiments conducted for event selection (Group 1), we established that the presence of *HaHB4* produced (i) no effect on the number of days to R1 (data not shown), (ii) a slight delay on days to R7 (data not shown), (iii) increased SY (Fig. 1A) due to increased seed numbers (Fig. 1B), and (iv) decreased individual seed weight (Fig. 1C). No event expressing *HaHB4* differed from the WT in days to R1 (i.e. beginning bloom). Across experiments, the WT took 42.48±9.20 days to reach stage R1, whereas the fastest event (b10H) took 42.34±9.21 days and the slowest event (a5H) took 42.77±8.96 days (i.e. a mean of only 0.43 d between the slowest and the fastest cultivars). By contrast, all events expressing *HaHB4* tended to show delayed senescence compared with the WT, although the number of days to R7 (i.e. beginning maturity) was slightly altered among them (maximum range of 1.66 days across mean values). The difference from the WT (mean 113.9±10.4 d to R7) was significant (*P*<0.05) only for the a5H event (mean 115.6±10.7 d to R7), and final harvest was done on the same date for all genotypes. The trade-off between increased seed numbers and decreased seed weight was only partial for b10H (SY larger than the SY of W82) and total for the other events (SY equal to the SY of W82), and consequently b10H was selected for subsequent studies.

### Transgenic soybean significantly outyields its control in field trials

**Fig. 2.**
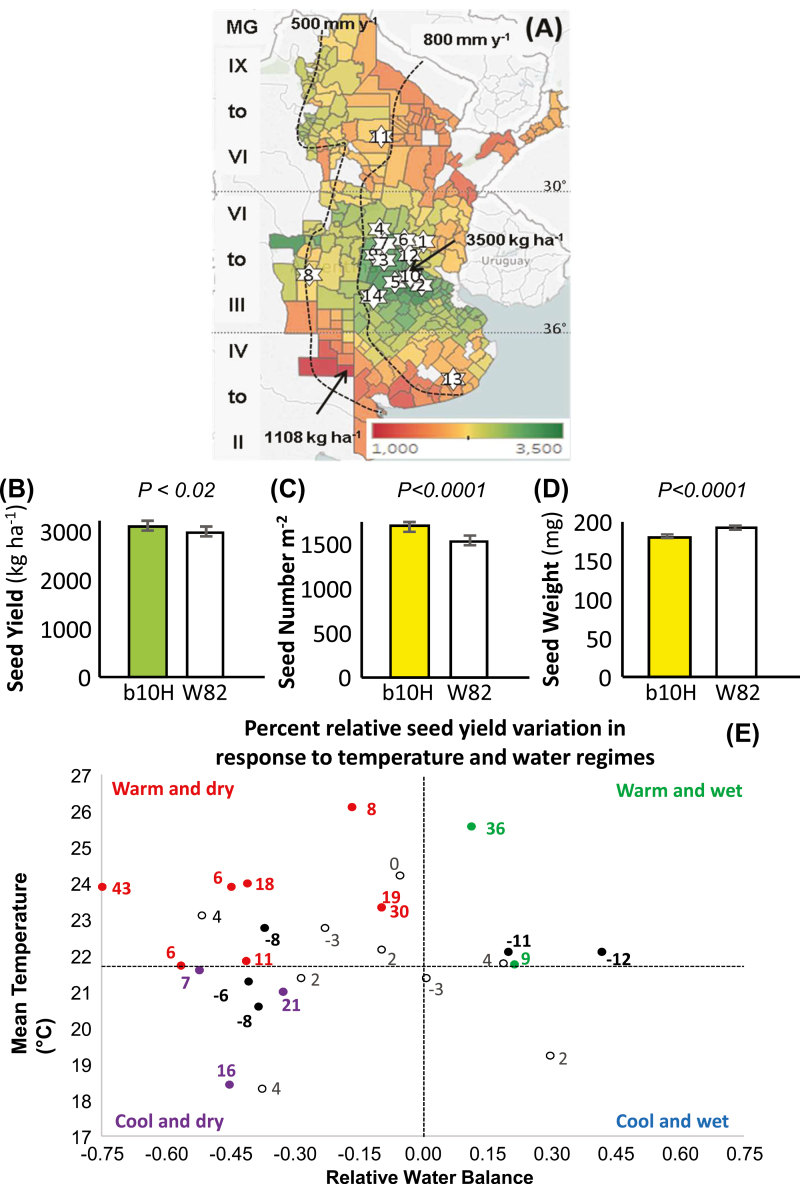
Transgenic event b10H outyields the wild-type cultivar W82 across 27 field-based experiments, particularly in warm and dry environments. (A) Location of the 14 sites corresponding to the experimental network of INDEAR (details in Supplementary Table S1). The colored scale in the map represents the 22-year mean seed yield of soybeans for the period between 1996 (the year of release of Roundup Ready cultivars) and 2018 in Argentina. Non-colored areas correspond to counties with fewer than 10 records for the evaluated period, and arrows indicate the counties with maximum (3500 kg ha^−1^) and minimum (1108 kg ha^−1^) mean seed yields. Roman numerals on the left describe the currently recommended maturity groups (MG) across the latitude gradient. Dashed lines indicate the 500 and 800 mm year^−1^ isohyets. (B–D) Mean values of seed yield (B), seed number m^−2^ (C), and individual seed weight (D) of b10H and W82 across 27 experiments. Error bars represent SEM×2. (E) Relationship of mean temperature and mean relative water balance of each experiment (*n*=27; Supplementary Table S1). Dashed lines represent the median mean temperature (horizontal) and the null relative water balance (vertical). The values next to each symbol represent the relative seed yield [RSY=(SY_TG_−SY_WT_)/SY_WT_; SY_TG_: seed yield of TG event b10H; SY_WT_: seed yield of WT parental cultivar W82]. Different colors represent cases with (i) RSY≥0.05 (SY_TG_>SY_WT_), in filled non-black symbols that identify the environmental group represented in each quadrant, (ii) RSY≤–0.05 (SY_WT_>SY_TG_), in filled black symbols, and (iii) –0.05<RGY<0.05 (SY_TG_≈SY_WT_), in open symbols.

From the results of 27 field experiments performed across a wide range of environmental conditions (Fig. 2A), it could be established that the TG cultivar b10H significantly outyielded the parental WT cultivar W82 (Fig. 2B). This advantage averaged +4.05% (range –11% to +43%) and held across the whole environmental range explored (Supplementary Fig. S1), which extended from a minimum of 1540 kg ha^−1^ to a maximum of 4540 kg ha^−1^. Models fitted to the response of each genotype to the environmental index indicated that b10H outyielded W82 across all environments with seed yield lower than 4898 kg ha^−1^. This threshold was never met in any of the evaluated environments. The SY advantage of b10H was a result of the larger number of seeds produced (mean +10.6%; Fig. 2C), which was not compensated by the observed reduction in individual seed weight (mean –6.5%; Fig. 2D). No crossover interaction was detected for SY components across environmental indexes (Supplementary Fig. S1), being b10H>W82 for the main determinant of SY (i.e. seed number m^−2^; Fig.1B). For both cultivars, final SY was tightly related to seed numbers (*r*^*2*^≥0.856; *P*<0.001) and to a much lesser extent to individual seed weight (*r*^*2*^≤0.086; 0.01<*P*<0.10).

When environments were sorted into four groups depending on the combination of mean temperature and RWB along the cycle (Fig. 2E), it could be observed that the majority of the experiments (13 cases) fell into the warm and dry category (i.e. RWB ≤0 and mean temperature ≥22ºC), followed by cool and dry (7 cases), then warm and wet (5 cases), and finally cool and wet (2 cases). In dry environments (i.e. RWB ≤0), the mean relative SY (RSY) was +8.6% (i.e. TG>WT). Within this group, the warm and dry subgroup had a mean RSY of +10.5%, whereas the dry and cool subgroup had a mean RSY of +5.1%. The mean RSY of wet environments was +3.6%, being +5.2% for the warm and wet subgroup and almost null (–0.5%) for the cool and wet subgroup. Although wet and cool environmental conditions are not preponderant among common growing conditions experienced by soybean crops, further evaluation is necessary in order to test the efficacy of the TG event under wet and cool conditions. It is important to highlight that cases with negative RSYs were scattered across all environmental categories. Therefore, negative values could not be attributed to a specific environmental condition but to some other factor(s) that caused *HaHB4* to be less effective.

### Differential traits between transgenic and wild-type soybean grown in the greenhouse

**Fig. 3.**
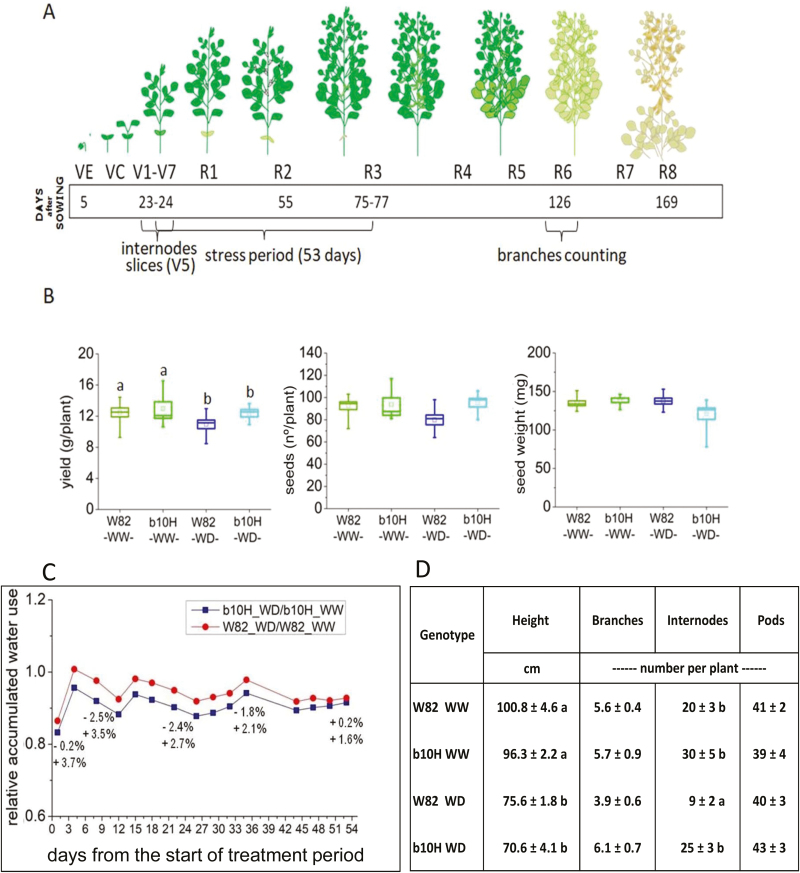
Transgenic soybean cultivar b10H grown in the greenhouse under contrasting watering regimes differs from its control W82 in seed yield and its components. (A) Schematic representation of soybean developmental stages and dates when the drought stress treatment was applied, as well as dates when data were collected. (B) Comparison of seed yield and its components (seed number and individual seed weight) per plant in well-watered (WW) and water-deficit (WD) plants; water deficit was applied in the period indicated as “stress period” in (A). The range of the box plots represents SEM×1. Horizontal lines in the box plots represent the 50th percentile, and the inner square represents the mean value. The vertical line represents the recorded minimum and maximum values. Different letters above the box plots indicate significant differences between samples (*P*<0.05). (C) Relative cumulative water use during the treatment period. Numbers next to some symbols (in %) illustrate the relative variation in cumulative water use between W82 and b10H, computed as $x=\frac{b10H-W82}{W82}*100$. Upper numbers are for WD plants and lower ones are for WW plants. (D) Comparison of plant height and the number of branches, internodes, and pods between treatments described in (B). Data are mean ±SE; means with the same letter within a column do not differ at *P*<0.05.

In order to understand which physiological traits could be responsible for the drought/warm-tolerant phenotype observed in *HaHB4* TG plants, a morpho-physiological evaluation was performed on plants grown in the greenhouse under well-watered or water-deficit conditions (Fig. 3A). TG b10H plants exhibited a trend towards increased SY in both conditions, even though the SY of both genotypes was significantly affected (*P*<0.05) by water deficit (Fig. 3B). Differences in SY were accompanied by similar trends in seed numbers but not in seed weight (Fig. 3B). Relative water use of water-deficit plants was smaller for the TG than for the WT (Fig. 3C), but this response was mainly a consequence of the enhanced water use of the TG cultivar under well-watered conditions (Fig. 3C). TG plants tended to be shorter and to have more branches, internodes, and pods per plant than W82 plants (Fig. 3D).

**Fig. 4.**
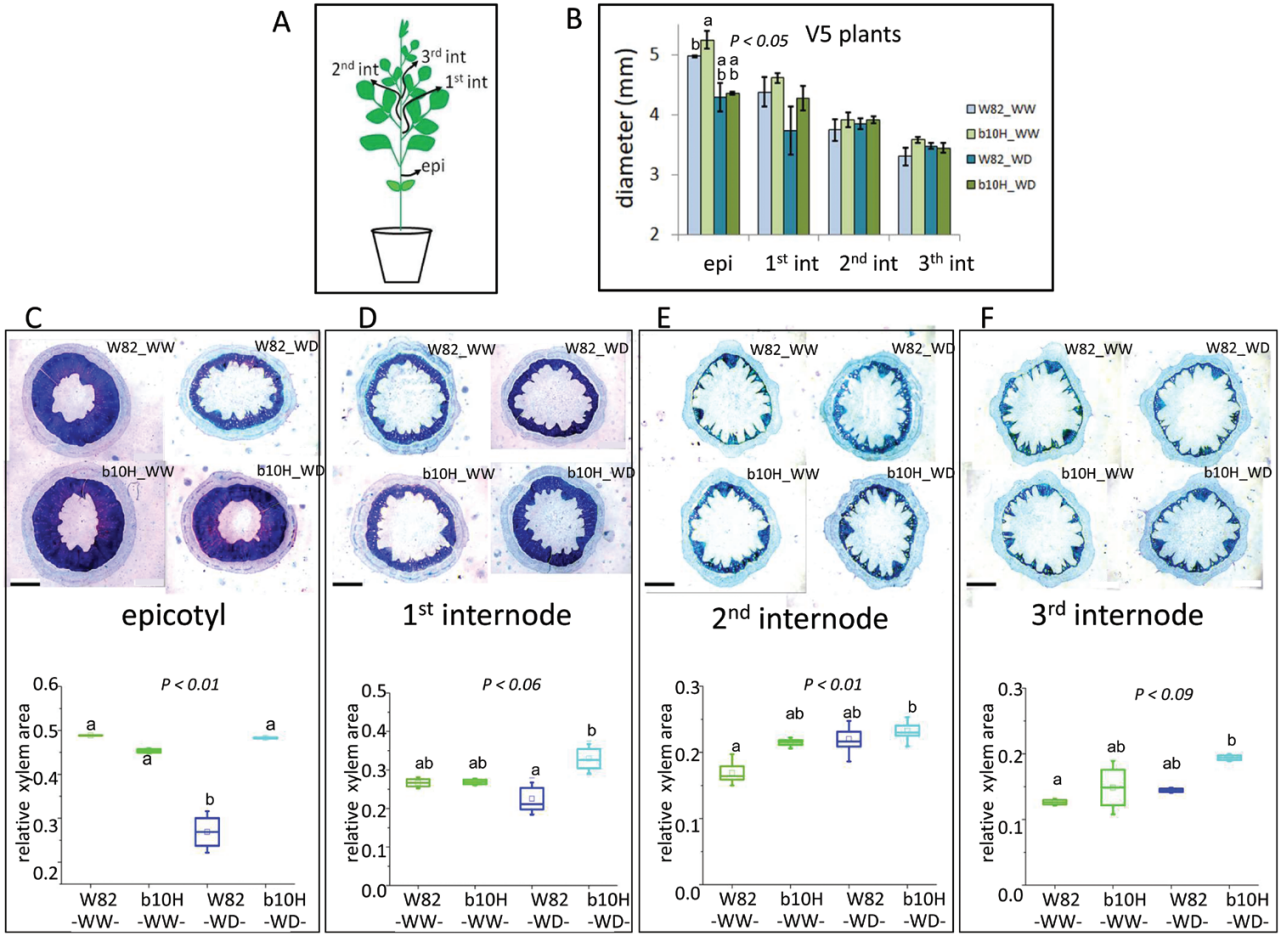
Transgenic soybean plants exhibit a wider stem diameter and larger xylem area than controls. (A) Schematic representation of a soybean plant indicating the stem sections that were harvested for morphological analyses. (B) Stem diameter of stage V5 plants in well-watered (WW) and water-deficit (WD) plants of TG b10H and WT W82. Data are mean ±SE. (C–F) Histological epicotyl (C), hypocotyl (D), and stem (E, F) cross-sections stained with safranin/fast green (upper panel) and quantification of relative xylem area (lower panel). Total internodes, xylem area, and xylem relative area were plotted and calculated using 3–4 biological replicates. Bar =1 mm. Detail of box plots in C–F is as described in Fig. 3. Different letters indicate significant differences according to Tukey comparisons (*P*<0.05).

Measurements of the diameters of epicotyls and first, second, and third internodes (Fig. 4A) indicated that these structures of b10H tended to be wider than those of W82 in stressed plants (Fig. 4B). The same trend was observed for xylem area, whereas all these characteristics differed less markedly in well-watered plants (Fig. 4C–FSupplementary Fig. S2).

### Plant phenotyping in field trials indicates significant differences between transgenic b10H and controls

**Fig. 5.**
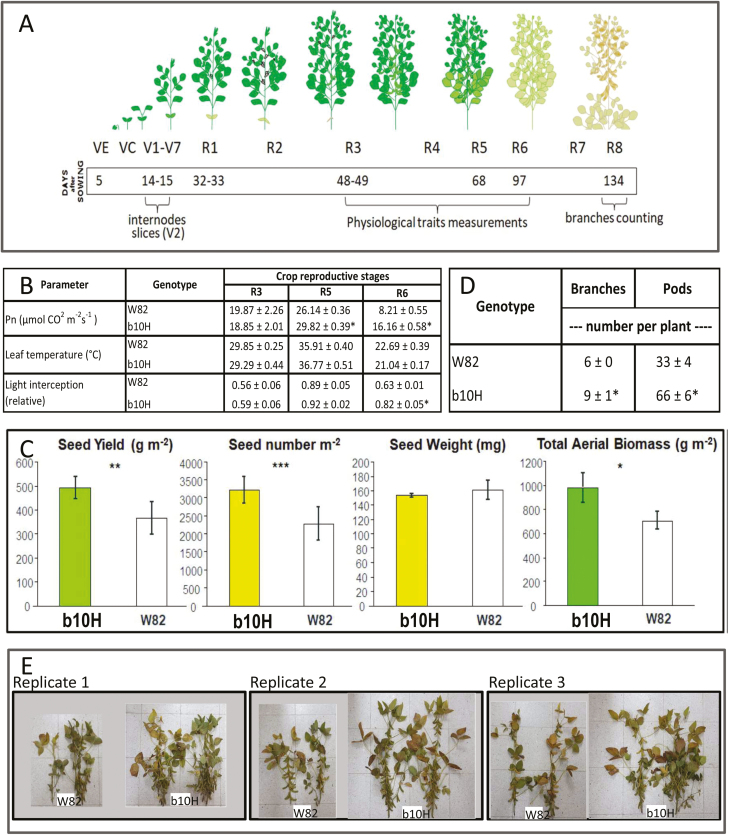
Seed yield components differ between field-grown transgenic b10H and wild-type W82 soybean in the warm and wet environment of the IAL site. (A) Details of assessed characteristics and dates of data collection during the soybean crop cycle in a field trial carried out at the IAL site. (B–D) Comparison between b10H and W82 of (B) physiological traits measured at different growth stages (Pn, net photosynthetic rate), (C) seed yield and yield components, and (D) number of branches and pods per plant. (E) Illustrative pictures of plants collected from all replicates. In (B) and (D), data are mean ±SE and asterisks indicate significant differences between b10H and W82 (*P*<0.05). In (C), error bars represent SEM×2 and asterisks indicate significant differences between b10H and W82 (*P<0.10, **P<0.05, *** *P*<0.01).

**Fig. 6.**
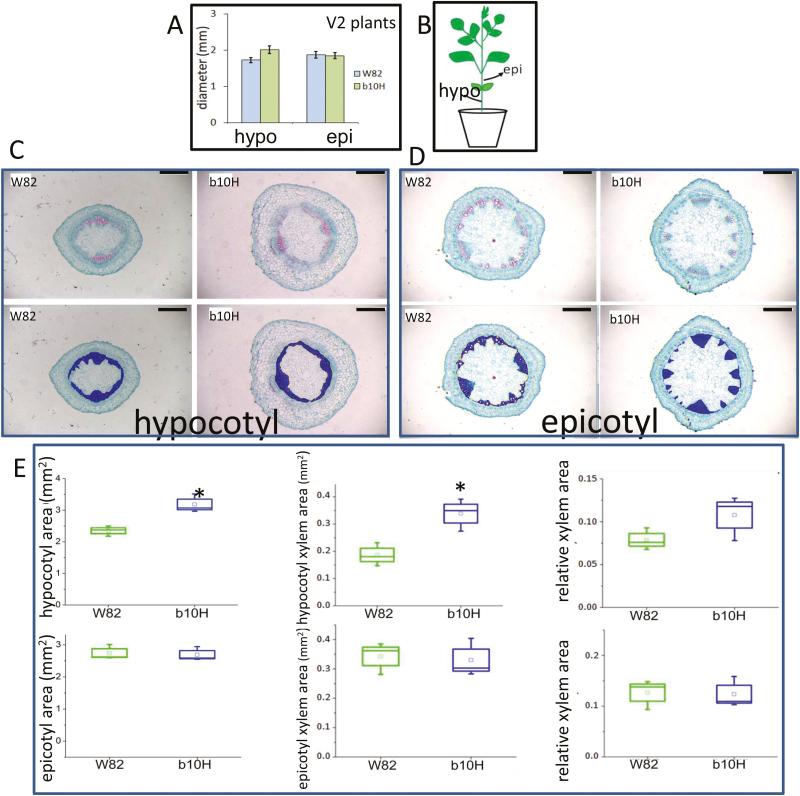
Stem and xylem diameters differ between transgenic b10H and wild-type W82 soybean plants grown at the IAL site. (A) Width of the hypocotyl (hypo) and epicotyl (epi) in stage V2 plants. Data are mean ±SEM. (B) Schematic representation of a soybean plant indicating the stem sections that were harvested for morphological analyses. (C, D) Illustrative pictures with standard (upper panel) or highlighted (bottom panel) xylem area of stem cross-sections obtained after safranin/fast green staining. Bars=0.5 mm. (E) Box plots of the cross-sectional areas of stem hypocotyl (upper panel) and epicotyl (bottom panel). Total internodes, xylem area, and xylem relative area were plotted and calculated using three biological replicates. Detail of box plots is as described in Fig. 3. Asterisks indicate significant differences according to Tukey comparisons (*P*<0.05).

To investigate whether the differential architectural and physiological traits observed in the greenhouse were conserved in plants grown in the field, production traits and several physiological traits were assessed in TG b10H and WT W82 soybeans grown in field experiments at the IAL site during 2017–2018 (Fig. 5A). Plants were irrigated but experienced some above-optimum temperatures (i.e. heat stress) during the cycle (Supplementary Table S1). No significant differences in the evaluated traits were detected between genotypes at R3. By contrast, b10H plants had a significantly (*P*<0.05) higher photosynthetic rate than W82 at R5 and R6 (Fig. 5B); a similar trend was also observed for light interception during seed filling (R6) and crop biomass (Fig. 5C). Differences in crop biomass were accompanied by significantly (*P*<0.05) increased SY and seed numbers in b10H (Fig. 5C). The greater seed numbers over-compensated for the reduction in individual seed weight (Fig. 5C). The differences in seed numbers were driven by the higher number of branches and pods in b10H compared with W82 plants (Fig. 5D, E). Finally, and similar to the phenotype observed in the greenhouse experiment, hypocotyl diameter and xylem area were larger in b10H than in W82 (Fig. 6Supplementary Fig. S3).

**Fig. 7.**
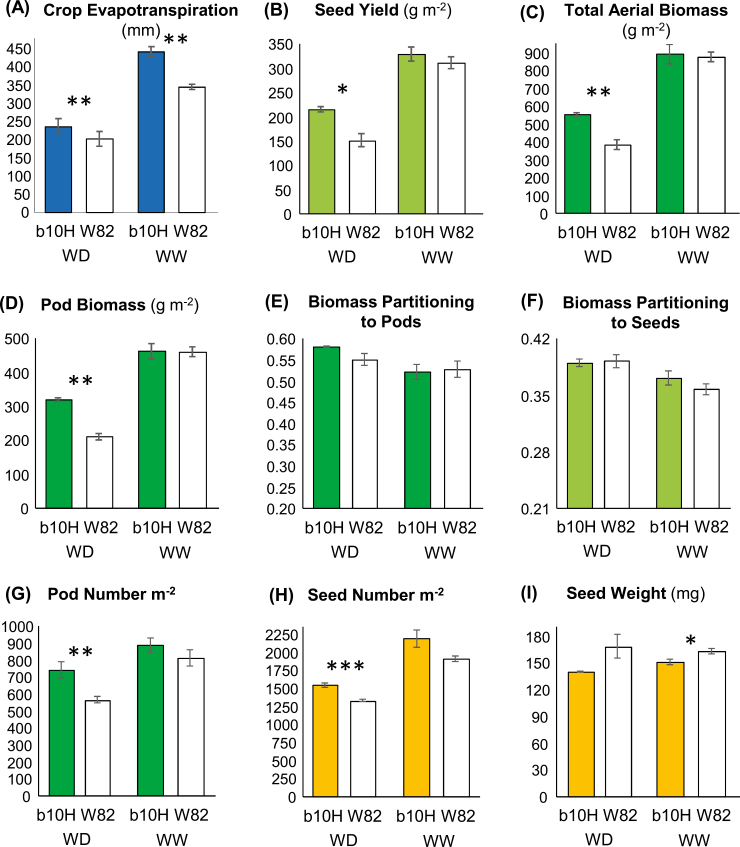
Field-based physiological analysis of soybean seed yield under contrasting water regimes. Traits were evaluated at harvest for TG cultivar b10H and WT cultivar W82 grown under water-deficit (WD) and well-watered (WW) conditions at the Pergamino site. (A) Crop evapotranspiration during the cycle. (B) Seed yield. (C) Total aerial biomass. (D) Pod biomass. (E) Biomass partitioning to pods (ratio of pod biomass to total aerial biomass). (F) Biomass partitioning to seeds or harvest index (ratio of seed yield to total aerial biomass). (G) Pod numbers. (H) Seed numbers. (I) Individual seed weight. Data are mean ±SEM and asterisks indicate significant differences between b10H and W82 (*P<0.10, **P<0.05, *** *P*<0.01).

Also during 2017–2018, when summer crops in the temperate region of Argentina were exposed to a severe drought caused by a La Niña phase of the El Niño–Southern Oscillation (ENSO) phenomenon, a field-based analysis of SY determination under two contrasting water regimes (water deficit and well-watered) was performed at the Pergamino site. Rainfall exclusion plus differential irrigation produced a large contrast in total ET_C_ between water-deficit and well-watered plots (Fig. 7A). A soil water survey included the topmost 185 cm and was performed from 23 d after sowing to stage R7. In both conditions, the water use of the TG cultivar b10H was higher (*P*<0.05) than that computed for the WT cultivar W82 (17.3% in water-deficit and 27.2% in well-water conditions). The physiological analysis indicated that b10H outyielded W82 under water deficit (43.4%), with no yield penalty under well-watered conditions (Fig. 7B). As observed in the IAL experiment, the increased SY measured in b10H compared with W82 under water deficit was driven by increased crop biomass (44.5%; Fig. 7C) and pod biomass (52.6%; Fig.7D) as well as by increased pod numbers (73.3%; Fig. 7G) and seed numbers (78.9%; Fig. 7H). Water deficit produced no significant difference between cultivars in the partitioning of biomass to pods (Fig. 7E) or to seeds (Fig. 7F), whereas individual seed weight in this condition was greater for W82 than for b10H (24.6%; Fig. 7I). Based on trends recorded for ET_C_ and production traits, a remarkably higher (≥22%) WUE was computed for TG than for WT cultivars exposed to water deficit. This trend held for biomass (WUE_B,ETc_ 2.3 g m^−2^mm^−1^ for the TG and 1.9 g m^−2^ mm^−1^ for W82) and for seed WUE (WUE_SY,ETc_ 0.91 g m^−2^ mm^−1^ for the TG and 0.74 g m^−2^ mm^−1^ for W82).

### Different molecular pathways are altered in transgenic soybean expressing HaHB4

**Fig. 8.**
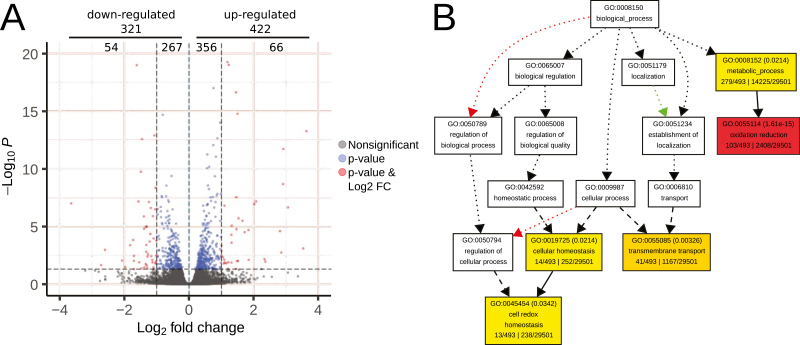
Differentially expressed genes (DEGs) in soybean cultivar b10H compared with wild-type W82 in field trials. (A) Volcano plot of DEGs determined by RNA-seq in TG b10H versus WT W82 plants. DEGs had a false discovery rate adjusted *P*-value <0.05 as indicated (horizontal cutoff). Additionally, genes above or below an absolute log_2_-fold change (FC) of 1 are shown in different colors and their numbers are given at the top of the plot. A few genes were omitted as their absolute fold change was greater than the chosen axis graph limits. (B) Directed acyclic graph of gene ontology terms including enriched categories of the biological process type.

Using RNA-seq of TG versus WT plants, we identified 743 DEGs (false discovery rate adjusted *P*<0.05; Fig. 8A, Supplementary Table S2), of which 120 presented an absolute log_2_-fold change >1. An inspection of the DEGs based on potential orthologous genes from Arabidopsis showed that there were genes previously associated to the heterologous expression of *HaHB4*, such as genes encoding lipoxygenases, trypsin inhibitors (Manavella *et al.*, 2008*b*), and the Cu/Zn superoxide dismutase CSD1 (Manavella *et al.*, 2006). There were also many DEGs related to heat, such as the homologues of genes encoding heat shock proteins AT-HSC70-1 (AT5G02500), AT-HSFB2A (AT5G62020), and Hsp81.4 (AT5G56000), and the homologue of the heat-related gene *HOT5* (AT5G43940, also known as *GSNOR*; Lee *et al.*, 2008).

A gene ontology enrichment analysis on all DEGs revealed “oxidation reduction”, “cell redox homeostasis”, and “transmembrane transport” as interesting and significantly enriched biological process terms (*P*<0.05; Fig. 8B, Supplementary Table S3). Among molecular function terms (Supplementary Table S3), some more descriptive categories were enriched, such as “protein disulfide oxidoreductase activity”, “iron ion binding”, “metal ion binding”, and “peroxidase activity” (Supplementary Table S3).

## Discussion

Second-generation TG crops (i.e. those with improved abiotic stress tolerance) have not reached the market yet, with the sole exception of drought-tolerant maize (http://www.isaaa.org/gmapprovaldatabase/) transformed with the bacterial RNA chaperons CspB and CspA (Castiglioni *et al.*, 2008). Besides the difficult and long regulatory processes that TG crops must go through, an additional constraint for this second generation is the non-universal nature of abiotic stresses, a characteristic that contrasts with the qualitative nature of biotic-oriented TG crops such as the emblematic RR soybeans and Bt maize. This constraint applies particularly to drought, the effects of which may be apparent as a broad spectrum of alternatives derived from multiple combinations of growth stages, intensities, and durations along the cycle (Chapman *et al.*, 2000; Tardieu, 2012).

Although there is a vast literature about drought-tolerant TG plants, mostly demonstrated in model plants and in controlled conditions (Skirycz *et al.*, 2011; Passioura 2012), it is possible that the huge investments required to commercially release drought-tolerant crops mean that efforts to develop such crops were aborted at different stages. Hence, the lack of drought-tolerant crops on the market is likely caused by experimental failures experienced when technologies tested in model plants and controlled conditions were assessed in field conditions.

HaHB4 is a sunflower TF whose expression is up-regulated by water deficit (Gago *et al.*, 2002). Its ectopic expression in Arabidopsis leads to drought-tolerant plants as a result of complex physiological mechanisms that do not include stomatal closure, a typical plant response to deal with water deficit involving a decrease in ethylene sensitivity (Manavella *et al.*, 2006). It was recently demonstrated that *HaHB4* was also able to confer drought tolerance to wheat in field trials (Gonzalez *et al.*, 2019), despite the large evolutionary distance between sunflower, Arabidopsis, and wheat. In this work we demonstrated that soybeans transformed with *HaHB4* also performed better than a WT control in stress-prone field conditions, particularly in warm and dry environments. These results were especially interesting because the expected drought-tolerant phenotype observed in Arabidopsis and wheat was expanded to a drought/heat-tolerant phenotype in soybean, which is a promising outcome for future climatic scenarios (IPCC, 2014).

Many independent TG events were obtained at the beginning of the research, including several with the combination of the *HaHB4* own promoter and the first intron of the Arabidopsis *COX5c* gene acting as an enhancer (Curi *et al.*, 2005; Cabello *et al.*, 2007). However, after several field trials in different environments, the most robust events were a5H and a11H (HaHB4 expression driven by the *35S CaMV* promoter) and b10H (expression driven by the *HaHB4* promoter). Such events tended to outyield the WT parental cultivar in all trials. Among them, b10H was the best yielding, and further studies were carried out only with this event.

Variation in SY among different water regimes and temperatures strongly suggested that the best target environments for TG b10H soybeans are warm and dry environments, in which it clearly outyielded controls (Fig. 2). Notably, the best performances were obtained in the drought experiment at the Pergamino site and the irrigated experiment at the IAL site, both of which were conducted during the La Niña phase of the ENSO phenomenon that took place during 2017–2018 (https://origin.cpc.ncep.noaa.gov), which brought below-normal rainfall together with episodes of above-optimum temperatures during the cycle of summer crops in the Pampas region of Argentina (Supplementary Table S1).

In all cases in which the TG outyielded the control, the response was associated with increased seed numbers that over-compensated for the decrease in individual seed weight. These characteristics (i.e. a partial trade-off between SY components) were also observed in the greenhouse experiment. Collectively, these results highlight the importance of improved crop growth (i.e. resource acquisition and allocation to reproductive organs) during the critical period for seed establishment (Vega *et al.*, 2001), as well as of the necessary improvement in crop photosynthetic capacity during seed filling (Borrás *et al.*, 2004) to avoid the trade-off between SY components. The SY history of soybean over the past 90 years has been recently reviewed, and potential targets to achieve yield improvement were proposed (Ainsworth *et al.*, 2012). As for cereals (Slafer *et al.*, 2015), optimization of carbon utilization/delivery to avoid flower abortion (i.e. improved fruiting efficiency) is among the targets in soybean (Egli and Bruening, 2002; Kantolic and Slafer, 2005), for which Ainsworth *et al.* (2012) proposed advancements in molecular breeding techniques aimed at the regulation of flower initiation and abortion. In this sense, TG b10H plants seem a promising genetic resource for future studies.

A notable feature of the TG b10H plants was their enhanced water use under well-watered conditions, particularly in field-grown plots (Fig. 7A). Because there was no evident difference between the phenotype of WT and TG plants under well-watered conditions (i.e. they had identical visual canopy characteristics), differences cannot be ascribed to a contrasting participation of soil evaporation in total crop water use, and can only be linked to enhanced transpiration of the TG plants. This difference may be ascribed to the increased xylem area and stem diameter observed in the TG plants; these traits may contribute to increased hydraulic conductivity and water use by crops (Richards and Passioura, 1981) and have been recently associated with increased yield of Arabidopsis and of sunflower plants (Cabello and Chan, 2019). However, differences in water use between TG and WT cultivars declined markedly (Fig. 7A) or almost disappeared (Fig. 3C) under water deficit, suggesting that the benefits observed in well-watered environments may have been partially or totally compensated in response to drought, probably by increased stomatal control of gas exchange in b10H compared with W82 (Sadock and Sinclair, 2009). Nevertheless, this control may have had a larger effect on water loss than on CO_2_ fixation (Liu *et al.*, 2005), a response supported by the pronounced increase in WUE observed under water deficit for b10H compared with W82. This response was not biased by differences related to the soil component of ET_C_, which was minimized in this growing condition. Collectively, these results are in good agreement with the slow-wilting soybean phenotype characterized by Fletcher *et al.* (2007), which might allow water conservation in drought conditions with no yield penalty (Devi *et al.*, 2014).

The acceleration of senescence by water stress during seed filling has been documented in soybean (de Souza *et al.*, 1997; Brevedan and Egli, 2003). Therefore, the delay in senescence reported here would be expected if the transgene promotes a reduced sensitivity to ethylene, as has been reported in Arabidopsis (Manavella *et al.*, 2006). The delayed senescence matched the delay in phenology recorded only for the R7 stage (i.e. late in the cycle), which could be visually assessed in several but not all field trials. More surprising was the tolerance of TG soybean to warm and dry growing conditions, which underscores the target environments for this technology. Such a response was not observed in either Arabidopsis-HaHB4 or field-tested wheat-HaHB4; in the case of the former because model plants analyzed for drought tolerance were always grown under controlled temperatures (Dezar et al., 2005*a*, *b*; Manavella *et al.*, 2006, 2008*a*, *b*) and never exposed to above-optimum temperatures, and in the case of the latter because the winter crop did not experience the combined effect of drought and high temperature during the cycle, except during seed filling in a few experiments (González *et al.*, 2019). Further investigations will be necessary to elucidate whether tolerance of warm and dry conditions is universal to all HaHB4-bearing species (i.e. it is gene specific) or is limited to soybean (i.e. there is a HaHB4 × species interaction).

Regarding the transcriptome of TG soybean, it is tempting to speculate that conserved mechanisms are displayed in different species, although further experimental evidence is needed to corroborate this hypothesis. This is because despite the great difference between 3-week-old culture chamber-grown Arabidopsis plants (Manavella *et al.*, 2006) and R5 soybeans grown in the field, remarkably, non-typically drought-responsive genes were observed to be differentially regulated, and several encoding lipoxygenases, trypsin inhibitors, and Cu/Zn superoxide dismutase appeared to be regulated in TG plants of both species. It was surprising to find heat shock-related genes differentially regulated in soybean, such as homologues of AT-HSC70-1, AT-HSFB2A, Hsp81.4, and HOT5 (Lee *et al.*, 2008); this finding supports the tolerance to high temperatures observed in the current work and will be investigated in the near future. Moreover, the gene ontology term “cell redox homeostasis”, which is known to be important under many stressful conditions (Vinocur and Altman, 2005), was enriched among DEGs. Future experiments will be aimed at defining whether such regulation persists under other environmental conditions or whether it is displayed by HaHB4 only when plants are subjected to warm and dry environments.

Finally, commercial varieties of soybean derived from the b10H event are currently being developed by multiple technology licensees. The event (named IND-00410-5 for regulatory and commercial release) has been conditionally approved for commercialization in Argentina in 2015 (https://www.argentina.gob.ar/agroindustria/alimentos-y-bioeconomia/ogm-comerciales), subject to Chinese import clearance for food and feed use (according to feed safety assessment principles; Parrott *et al.*, 2010), which is still pending. Brazil (https://news.agrofy.com.ar/noticia/181033/aprobaron-brasil-soja-hb4-tolerante-sequia) and more recently the USA (Alloatti *et al.*, 2017) have also approved the b10H event for production and consumption purposes. Together, these three countries represent ~80% of global soybean production. Elite varieties are currently being multiplied and a few thousand hectares are expected to go into production in the coming crop cycle in the Southern Hemisphere. The technology is expected to be broadly launched in South America in 2020–2021, under the HB4 brand.

## Supplementary data

Supplementary data are available at *JXB* online.

Table S1. General growing conditions along the cycle experienced by soybean crops in 27 experiments.

Table S2. Differentially expressed genes between transgenic and W82 control plants.

Table S3. GO terms analysis of 743 differentially expressed genes.

Fig. 1. Response of seed yield and seed yield components of W82 and b10H to their corresponding environmental indexes.

Fig. S2. Biological replicates of histological stem cuts of W82 and b10H transgenic plants grown in the greenhouse (replicates of images shown in Fig. 4C–F).

Fig. S3. Biological replicates of histological stem cuts of W82 and b10H plants grown in the field at the IAL site (2017–2018; replicates of images shown in Fig. 6C–D).

eraa064_suppl_supplementary_figures_S1_S3_table_S1

eraa064_suppl_supplementary_table_S2

eraa064_suppl_supplementary_table_S3

## References

[CIT0001] Ainsworth EA, Yendrek CR, Skoneczka JA, Long SP 2012 Accelerating yield potential in soybean: potential targets for biotechnological improvement. Plant, Cell and Environment 35, 38–52. 10.1111/j.1365-3040.2011.02378.x 21689112

[CIT0002] Alloatti J, Blackford C, Burachik M, et al 2017 Petition for determination of non-regulated status for the new plant variety HB4 soybean (IND-00410-5) intended for environmental release and food and feed use USDA (Application 17-223-01p) https://www.aphis.usda.gov/aphis/ourfocus/biotechnology/permits-notifications-petitions/petitions/petition-status

[CIT0003] Andrews S 2010 FastQC: a quality control tool for high throughput sequence data http://www.bioinformatics.babraham.ac.uk/projects/fastqc

[CIT0004] Arce AL, Raineri J, Capella M, Cabello JV, Chan RL 2011 Uncharacterized conserved motifs outside the HD-Zip domain in HD-Zip subfamily I transcription factors; a potential source of functional diversity. BMC Plant Biology 11, 42. 21371298 10.1186/1471-2229-11-42 PMC3060862

[CIT0005] Ariel FD, Manavella PA, Dezar CA, Chan RL 2007 The true story of the HD-Zip family. Trends in Plant Science 12, 419–426. 17698401 10.1016/j.tplants.2007.08.003

[CIT0006] Belamkar V, Weeks NT, Bharti AK, Farmer AD, Graham MA, Cannon SB 2014 Comprehensive characterization and RNA-Seq profiling of the HD-Zip transcription factor family in soybean (*Glycine max*) during dehydration and salt stress. BMC Genomics 15, 950. 25362847 10.1186/1471-2164-15-950 PMC4226900

[CIT0007] Bolger AM, Lohse M, Usadel B 2014 Trimmomatic: A flexible trimmer for Illumina Sequence Data. Bioinformatics 30, 2114–2120. 24695404 10.1093/bioinformatics/btu170 PMC4103590

[CIT0008] Borrás L, Slafer GA, Otegui ME 2004 Seed dry weight response to source–sink manipulations in wheat, maize and soybean: a quantitative reappraisal. Field Crops Research 86, 131–146.

[CIT0009] Brevedan RE, Egli DB 2003 Short periods of water stress during seed filling, leaf senescence, and yield of soybean. Crop Science 43, 2083–2088.

[CIT0010] Cabello JV, Chan RL 2019 Arabidopsis and sunflower plants with increased xylem area show enhanced seed yield. The Plant Journal 99, 717–732. 31009150 10.1111/tpj.14356

[CIT0011] Cabello JV, Dezar CA, Manavella PA, Chan RL 2007 The intron of the *Arabidopsis thaliana COX5c* gene is able to improve the drought tolerance conferred by the sunflower *Hahb*-4 transcription factor. Planta 226, 1143–1154. 17569080 10.1007/s00425-007-0560-9

[CIT0012] Castiglioni P, Warner D, Bensen RJ, et al 2008 Bacterial RNA chaperones confer abiotic stress tolerance in plants and improved grain yield in maize under water-limited conditions. Plant Physiology 147, 446–455. 18524876 10.1104/pp.108.118828 PMC2409020

[CIT0013] Chan RL, Gonzalez DH 2013 Modified *Helianthus annuus* transcription factor improves yield. Patent US20130263327 and WO2013126451.

[CIT0014] Chapman S, Cooper M, Hammer GL, Butler DG 2000 Genotype by environment interactions affecting grain sorghum. II. Frequencies of different seasonal patterns of drought stress are related to location effects on hybrid yields. Australian Journal of Agricultural Research 51, 209–221.

[CIT0015] Chen LM, Fang YS, Zhang CJ, et al 2019 *GmSYP24*, a putative syntaxin gene, confers osmotic/drought, salt stress tolerances and ABA signal pathway. Scientific Reports 9, 5990. 30979945 10.1038/s41598-019-42332-5 PMC6461667

[CIT0016] Cortes C, Vapnik V 1995 Support vector networks. Machine Learning 20, 273–297.

[CIT0017] Curi GC, Chan RL, Gonzalez DH 2005 The leader introns of *Arabidopsis thaliana* genes encoding cytochrome c oxidase subunit 5c promote high-level expression by increasing transcript abundance and translation efficiency. Journal of Experimental Botany 56, 2563–2571. 16061502 10.1093/jxb/eri250

[CIT0018] D’Ambrogio de Argueso A 1986 Manual de técnicas en histología vegetal. Buenos Aires: Hemisferio Sur.

[CIT0019] De Souza PI, Egli DB, Bruening WP 1997 Water stress during seed filling and leaf senescence in soybean. Agronomy Journal 89, 807–812.

[CIT0020] Devi JM, Sinclair TR, Chen P, Carter TE 2014 Evaluation of elite southern maturity soybean breeding lines for drought-tolerant traits. Agronomy Journal 106, 1947–1954.

[CIT0021] Dezar C, Fedrigo G, Chan RL 2005a The promoter of the sunflower HD-Zip protein gene *Hahb4* directs tissue-specific expression and is inducible by water stress, high salt concentrations and ABA. Plant Science 169, 447–459.

[CIT0022] Dezar CA, Gago GM, Gonzalez DH, Chan RL 2005b *Hahb-4*, a sunflower homeobox-leucine zipper gene, is a developmental regulator and confers drought tolerance to *Arabidopsis thaliana* plants. Transgenic Research 14, 429–440. 16201409 10.1007/s11248-005-5076-0

[CIT0023] Dobin A, Davis CA, Schlesinger F, Drenkow J, Zaleski C, Jha S, Batut P, Chaisson M, Gingeras TR 2013 STAR: ultrafast universal RNA-seq aligner. Bioinformatics 29, 15–21. 23104886 10.1093/bioinformatics/bts635 PMC3530905

[CIT0024] Egli DB, Bruening WP 2002 Flowering and fruit set dynamics at phloem-isolated nodes in soybean. Field Crops Research 79, 9–19.

[CIT0025] Ewels P, Magnusson M, Lundin S, Käller M 2016 MultiQC: summarize analysis results for multiple tools and samples in a single report. Bioinformatics 32, 3047–3048. 27312411 10.1093/bioinformatics/btw354 PMC5039924

[CIT0026] Fehr WR, Caviness CE 1977 Stages of soybean development. Special Report 87. Ames: Iowa State University.

[CIT0027] Fletcher AL, Sinclair TR, Allen LH Jr 2007 Transpiration response to vapor pressure deficit in well watered ‘slow wiliting’ and commercial soybean. Environmental and Experimental Botany 61, 145–152.

[CIT0028] Gago GM, Almoguera C, Jordano J, Gonzalez DH, Chan RL 2002 *Hahb-4*, a homeobox-leucine zipper gene potentially involved in abscisic acid-dependent responses to water stress in sunflower. Plant, Cell and Environment 25, 633–640.

[CIT0029] González FG, Capella M, Ribichich KF, Curín F, Giacomelli JI, Ayala F, Watson G, Otegui ME, Chan RL 2019 Field-grown transgenic wheat expressing the sunflower gene *HaHB4* significantly outyields the wild type. Journal of Experimental Botany 70, 1669–1681. 30726944 10.1093/jxb/erz037 PMC6411379

[CIT0030] Goodstein DM, Shu S, Howson R, et al 2012 Phytozome: a comparative platform for green plant genomics. Nucleic Acids Research 40, D1178–D1186. 22110026 10.1093/nar/gkr944 PMC3245001

[CIT0031] Hartman GL, West ED, Herman TK 2011 Crops that feed the world 2. Soybean-worldwide production, use, and constraints caused by pathogens and pests. Food Security 3, 5.

[CIT0032] Höfgen R, Willmitzer L 1988 Storage of competent cells for Agrobacterium transformation. Nucleic Acids Research 16, 9877. 3186459 10.1093/nar/16.20.9877 PMC338805

[CIT0033] IPCC. 2014 Climate Change 2014: Synthesis Report. Contribution of Working Groups I, II and III to the Fifth Assessment Report of the Intergovernmental Panel on Climate Change. Geneva: IPCC.

[CIT0034] Kantolic AG, Slafer GA 2005 Reproductive development and yield components in indeterminate soybean as affected by post-flowering photoperiod. Field Crops Research 93, 212–222.

[CIT0035] Köster J, Rahmann S 2018 Snakemake—a scalable bioinformatics workflow engine. Bioinformatics 34, 3600. 29788404 10.1093/bioinformatics/bty350

[CIT0036] Lee U, Wie C, Fernandez BO, Feelisch M, Vierling E 2008 Modulation of nitrosative stress by *S*-nitrosoglutathione reductase is critical for thermotolerance and plant growth in *Arabidopsis*. The Plant Cell 20, 786–802. 18326829 10.1105/tpc.107.052647 PMC2329944

[CIT0037] Li Y, Chen Q, Nan H, Li X, Lu S, Zhao X, Liu B, Guo C, Kong F, Cao D 2017 Overexpression of *GmFDL19* enhances tolerance to drought and salt stresses in soybean. PLoS One 12, e0179554. 28640834 10.1371/journal.pone.0179554 PMC5480881

[CIT0038] Li H, Handsaker B, Wysoker A, Fennell T, Ruan J, Homer N, Marth G, Abecasis G, Durbin R; 1000 Genome Project Data Processing Subgroup. 2009 The sequence alignment/map format and SAMtools. Bioinformatics 25, 2078–2079. 19505943 10.1093/bioinformatics/btp352 PMC2723002

[CIT0039] Liang C 2017 Genetically modified crops with drought tolerance: achievements, challenges, and perspectives. In: Hossain MA, Wani SH, Bhattachajee S, Burritt DJ, Tran L-SP, eds. Drought stress tolerance in plants, Vol 2: Molecular and genetic perspectives. Cham: Springer International Publishing Switzerland, 531–547.

[CIT0040] Liao Y, Smyth GK, Shi W 2014 featureCounts: an efficient general purpose program for assigning sequence reads to genomic features. Bioinformatics 30, 923–930. 24227677 10.1093/bioinformatics/btt656

[CIT0041] Liu F, Andersen MN, Jacobsen SE, Jensen CR 2005 Stomatal control and water use efficiency of soybean (*Glycine max* L. Merr.) during progressive soil drying. Environmental and Experimental Botany 54, 33–40.

[CIT0042] Love MI, Huber W, Anders S 2014 Moderated estimation of fold change and dispersion for RNA-seq data with DESeq2. Genome Biology 15, 550. 25516281 10.1186/s13059-014-0550-8 PMC4302049

[CIT0043] Maddoni G, Otegui ME 1996 Leaf area, light interception, and crop development in maize. Field Crops Research 48, 81–87.

[CIT0044] Manavella PA, Arce AL, Dezar CA, Bitton F, Renou JP, Crespi M, Chan RL 2006 Cross-talk between ethylene and drought signalling pathways is mediated by the sunflower Hahb-4 transcription factor. The Plant Journal 48, 125–137. 16972869 10.1111/j.1365-313X.2006.02865.x

[CIT0045] Manavella PA, Dezar CA, Ariel FD, Drincovich MF, Chan RL 2008a The sunflower HD-Zip transcription factor HAHB4 is up-regulated in darkness, reducing the transcription of photosynthesis-related genes. Journal of Experimental Botany 59, 3143–3155. 18603614 10.1093/jxb/ern170

[CIT0046] Manavella PA, Dezar CA, Bonaventure G, Baldwin IT, Chan RL 2008b HAHB4, a sunflower HD-Zip protein, integrates signals from the jasmonic acid and ethylene pathways during wounding and biotic stress responses. The Plant Journal 56, 376–388. 18643970 10.1111/j.1365-313X.2008.03604.x

[CIT0047] Manavella PA, Dezar CA, Chan RL 2008b Two ABREs, two redundant root-specific and one W-box *cis*-acting elements are functional in the sunflower *HAHB4* promoter. Plant Physiology and Biochemistry 46, 860–867. 18586510 10.1016/j.plaphy.2008.05.003

[CIT0048] Morran S, Eini O, Pyvovarenko T, Parent B, Singh R, Ismagul A, Eliby S, Shirley N, Langridge P, Lopato S 2011 Improvement of stress tolerance of wheat and barley by modulation of expression of DREB/CBF factors. Plant Biotechnology Journal 9, 230–249. 20642740 10.1111/j.1467-7652.2010.00547.x

[CIT0049] Parrott W, Chassy B, Ligon J, Meyer L, Petrick J, Zhou J, Herman R, Delaney B, Levine M 2010 Application of food and feed safety assessment principles to evaluate transgenic approaches to gene modulation in crops. Food and Chemical Toxicology 48, 1773–1790. 20399824 10.1016/j.fct.2010.04.017

[CIT0050] Passioura JB 2012 Phenotyping for drought tolerance in grain crops: when is it useful to breeders? Functional Plant Biology 39, 851–859. 10.1071/FP12079 32480835

[CIT0051] Pereira AA, Morales AMAP, Borém A, Loureiro ME 2011 Expressão de genes da subfamília HD-Zip I em soja submetida à seca. Pesquisa Agropecuaria Brasileira 46, 884–889.

[CIT0052] Perotti MF, Ribone PA, Chan RL 2017 Plant transcription factors from the homeodomain-leucine zipper family I. Role in development and stress responses. IUBMB Life 69, 280–289. 28337836 10.1002/iub.1619

[CIT0053] Rasband WS 1997–2018 ImageJ. Bethesda: US National Institutes of Health https://imagej.nih.gov/ij/.

[CIT0054] R Core Team. 2018 R: A language and environment for statistical computing. Vienna: R Foundation for Statistical Computing. https://www.R-project.org/.

[CIT0055] Riechmann JL 2002 Transcriptional regulation: a genomic overview. The Arabidopsis Book 2002, e0085. 10.1199/tab.0085 PMC3243377 22303220

[CIT0056] Richards RA, Passioura JB 1981 Seminal root morphology and water use of wheat II. Environmental effects. Crop Science 21, 249–252.

[CIT0057] Sadock W, Sinclair TR 2009 Genetic variability of transpiration response to vapor pressure deficit among soybean cultivars. Crop Science 49, 955–960.

[CIT0058] Schmutz J, Cannon SB, Schlueter J, et al 2010 Genome sequence of the palaeopolyploid soybean. Nature 463, 178–183. 20075913 10.1038/nature08670

[CIT0059] Skirycz A, Vandenbroucke K, Clauw P, et al 2011 Survival and growth of *Arabidopsis* plants given limited water are not equal. Nature Biotechnology 29, 212–214. 10.1038/nbt.1800 21390020

[CIT0060] Slafer GA, Elia M, Savin R, García GA, Terrile II, Ferrante A, Miralles DJ, González FG 2015 Fruiting efficiency: an alternative trait to further rise wheat yield. Food and Energy Security 4, 92–109.

[CIT0061] Somers DA, Samac DA, Olhoft PM 2003 Recent advances in legume transformation. Plant Physiology 131, 892–899. 12644642 10.1104/pp.102.017681 PMC1540289

[CIT0062] Tardieu F 2012 Any trait or trait-related allele can confer drought tolerance: just design the right drought scenario. Journal of Experimental Botany 63, 25–31. 21963615 10.1093/jxb/err269

[CIT0063] Tian T, Liu Y, Yan H, You Q, Yi X, Du Z, Xu W, Su Z 2017 agriGO v2.0: a GO analysis toolkit for the agricultural community, 2017 update. Nucleic Acids Research 45, W122–W129. 28472432 10.1093/nar/gkx382 PMC5793732

[CIT0064] Vega CRC, Andrade FH, Sadras VO, Uhart SA, Valentinuz OR 2001 Seed number as a function of growth. A comparative study in soybean, sunflower, and maize. Crop Science 41, 748–754.

[CIT0065] Vinocur B, Altman A 2005 Recent advances in engineering plant tolerance to abiotic stress: achievements and limitations. Current Opinion in Biotechnology 16, 123– 132. 10.1016/j.copbio.2005.02.001 15831376

[CIT0066] Wang N, Zhang W, Qin M, Li S, Qiao M, Liu Z, Xiang F 2017 Drought tolerance conferred in soybean (*Glycine max* L.) by GmMYB84, a novel R2R3-MYB transcription factor. Plant & Cell Physiology 58, 1764–1776. 29016915 10.1093/pcp/pcx111

