# Supplementary material for: Successful field performance in warm and dry environments of soybean expressing the sunflower transcription factor HB4
Source: J Exp Bot. 2020 Mar 6;71(10):3142–56. doi: 10.1093/jxb/eraa064 (PMC7260725; doi:10.1093/jxb/eraa064)
Supplement: eraa064_suppl_supplementary_figures_S1_S3_table_S1 [file eraa064_suppl_supplementary_figures_s1_s3_table_s1.pdf]

Successful field performance in dry-warm environments of soybean expressing the sunflower transcription factor HaHB4

Ribichich KF[a#], Chiozza M[b#], Ávalos-Britez S[cz], Cabello JV[az], Arce AL[az], Watson G[b], Arias C[d], Portapila M[d], Trucco F[b], Otegui ME[e*], Chan RL[a*]

Supplementary Figures 1-3 & Table S1

Supplementary Figure 1.
Response of seed yield and seed yield components of W82 and b10H to their corresponding environmental indexes

Comparison between the transgenic cv. b10H and the parental cv. W82 for seed yield (A), seed number (B), and individual seed weight (C) across all evaluated environments (Supplementary Table 1). The mean value of each environment corresponds to the average of all tested genotypes and for each trait is described as an environmental index (EI) . Fitted models in (A) differed at P<0.05 and indicated that b10H will outyield W82 across all environments with seed yield lower than 4898 kg ha^{-1}, a threshold never met in current research. No cross-over interaction was detected for models fitted to seed numbers (B) and seed weight (C). Ordinates of models fitted in (B) and (C) differed at P<0.0001.

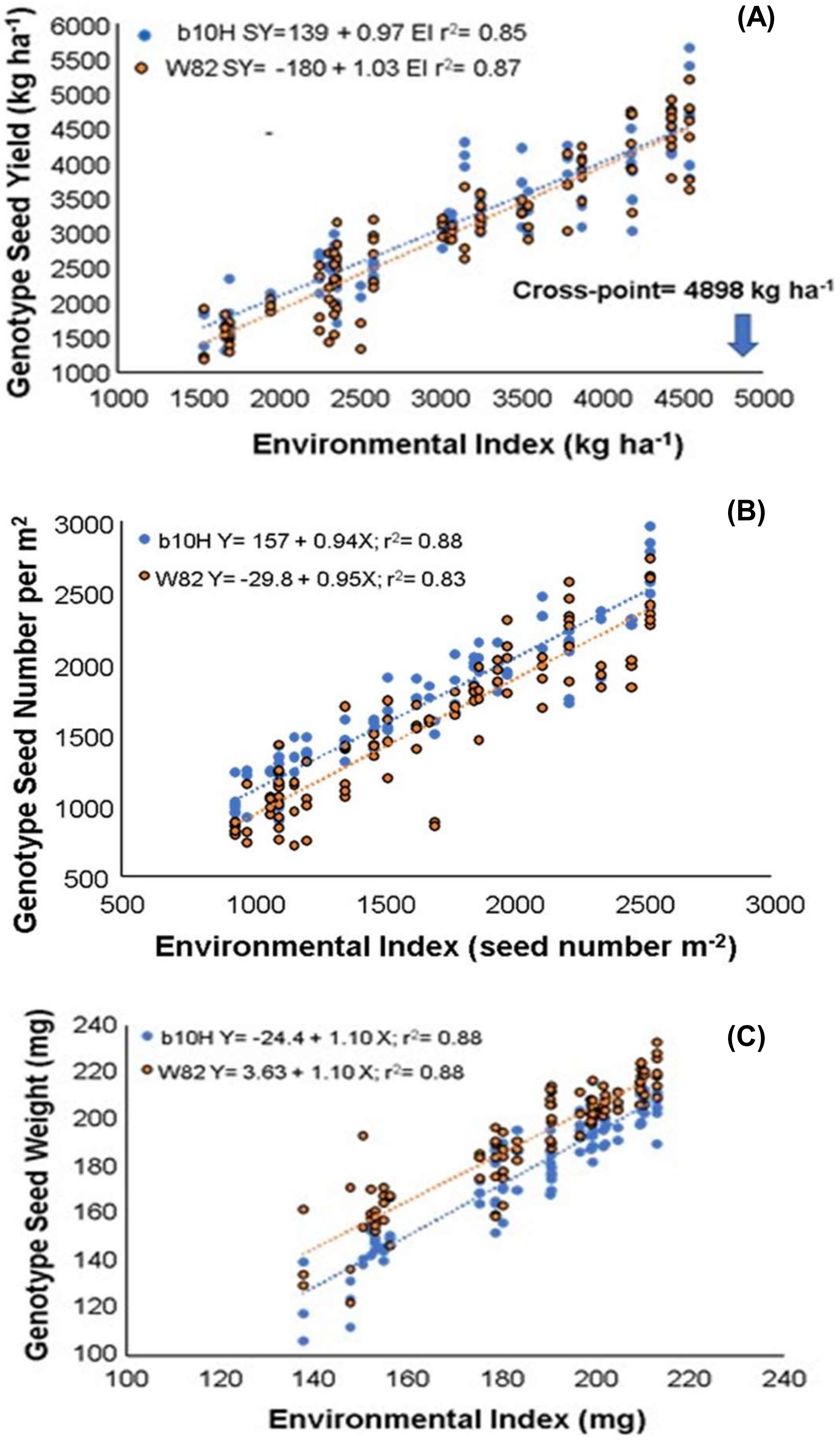

Supplementary Figure 2.

Biological replicates of histological stem cuts of W82 and b10H transgenic plants grown in the greenhouse (replicates of images shown in Figure 4C-F).

Stems of V5 plants in well-watered (WW) and water-deficit (WD) plants of TG b10H and WT W82. Internodes (epicotyls, 1st, 2nd and 3rd) slices stained with safranine-fast green (left images) and the same slices with highlighted xylem area (right panel). Bar length = 1 mm.

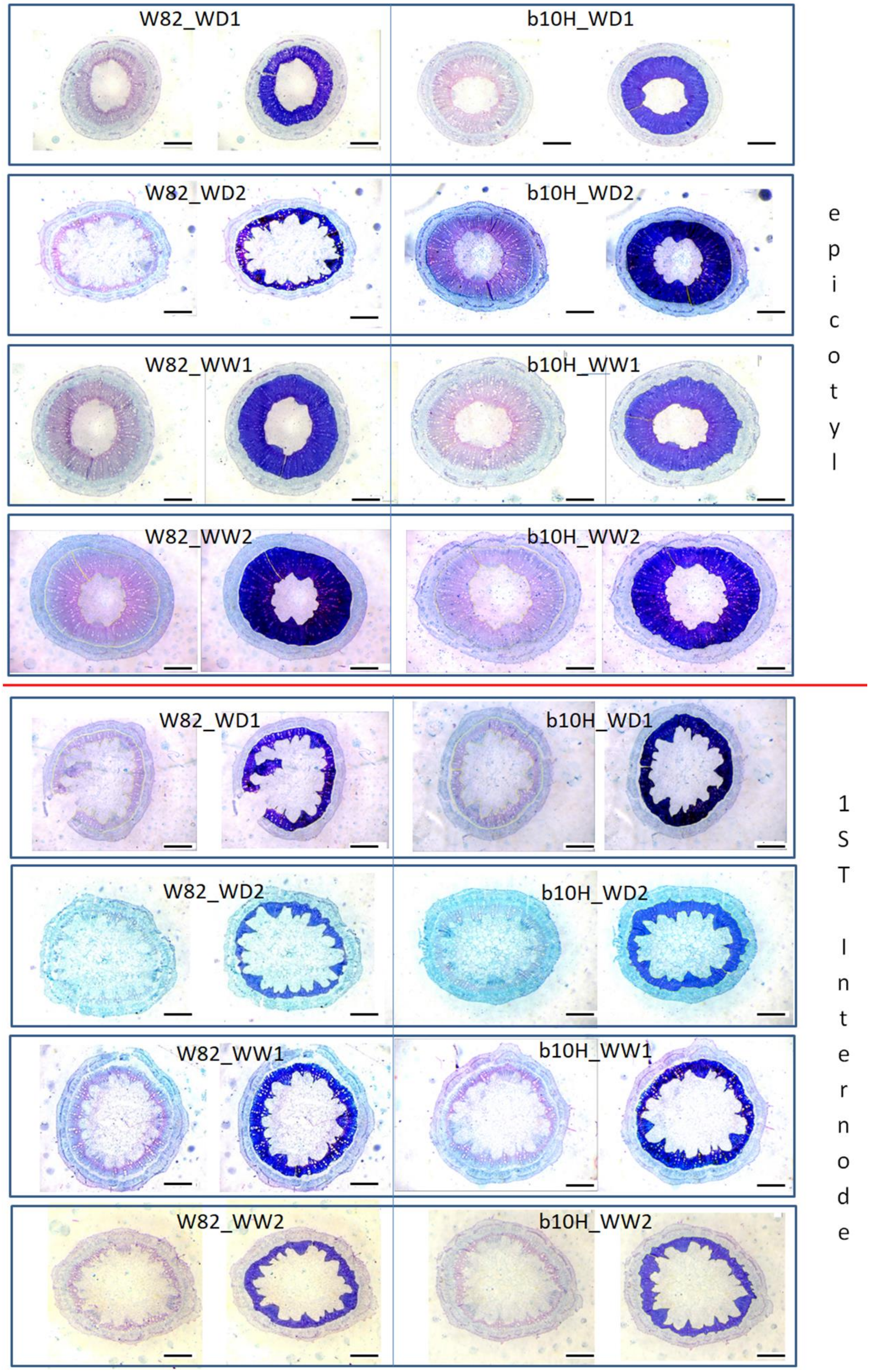

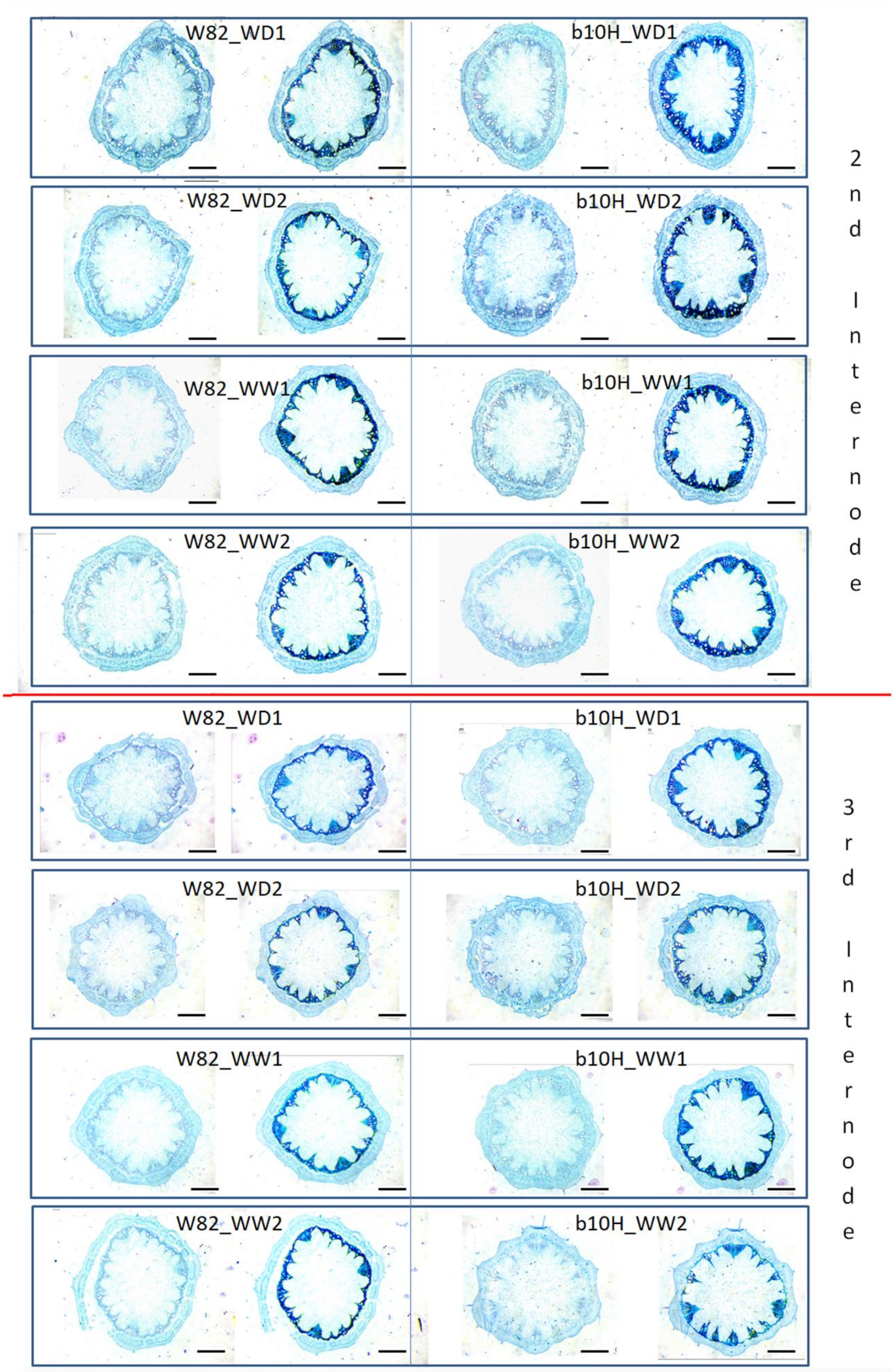
W82_WD1
b10H_WD1
W82_WD2
b10H_WD2
W82_WW1
b10H_WW1
W82_WW2
b10H_WW2
2nd Internode
W82_WD1
b10H_WD1
W82_WD2
b10H_WD2
W82_WW1
b10H_WW1
W82_WW2
b10H_WW2
3rd Internode

Supplementary Figure 3.
Biological replicates of histological stem cuts of W82 and b10H plants grown in the field at the IAL site (2017-2018; replicates of images shown in Figure 6C-D).
Stem (V2 hypocotyls and epicotyls) slices stained with safranine-fast green (left images) and the same slices with highlighted xylem area (right panel). b10H are transgenic plants and W82 are wild type controls. Bar length = 0.5 mm.

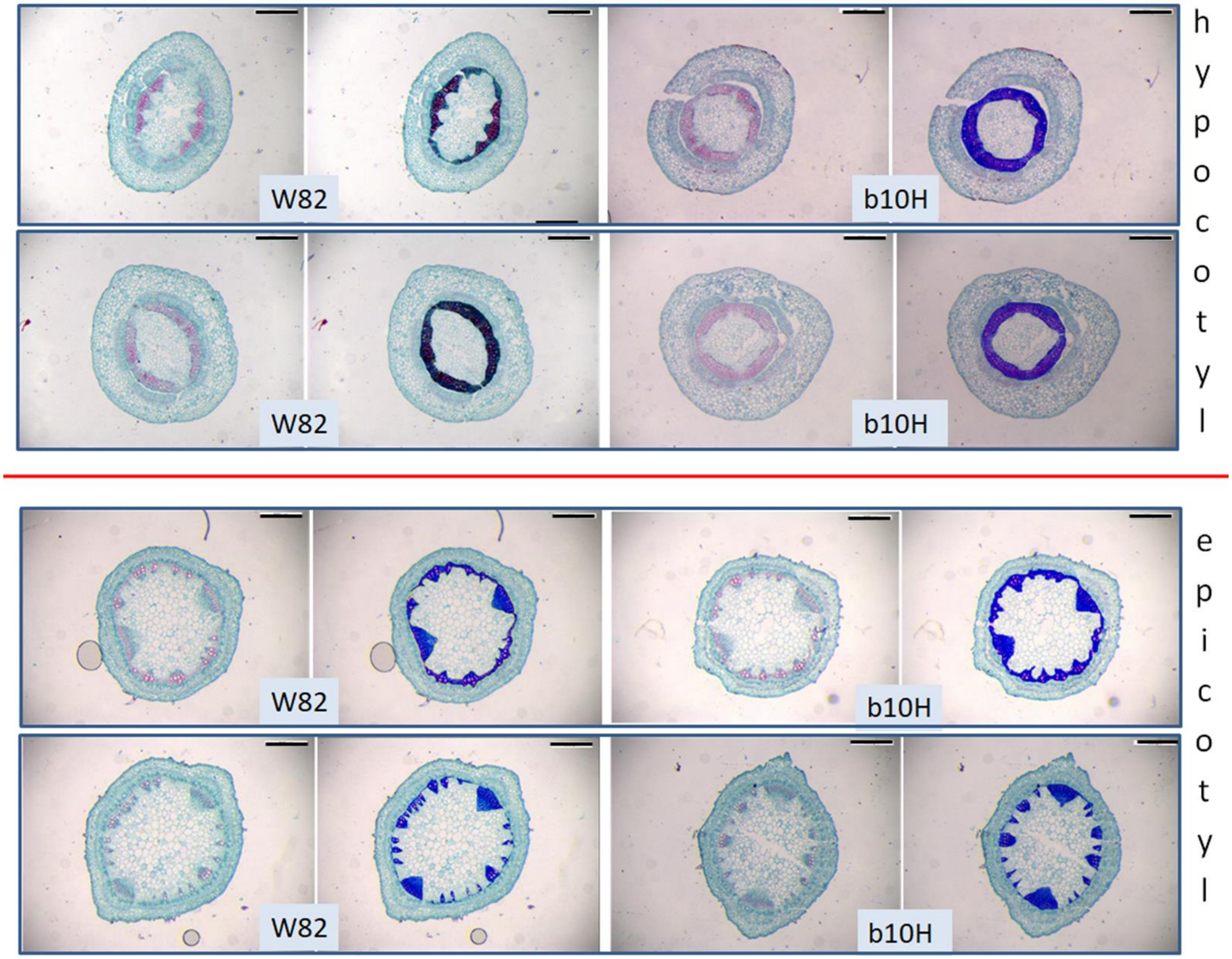

Supplementary Table 1. Description of experiments.

Data correspond to weather coditions experienced during the whole cycle by field-grown soybean crops in 27 experiments performed across 6 years and 14 sites of Argentina. Sites are indicated in the map of Fig. 2A. Different water regimes (IR: irrigated; R: rainfed; WD: water deficit with rain-out shelters), sowing dates (ES: early sowing; DS: delayed sowing), and phosphorus fertilizer rates (MAP: monoammonium phosphate, in kg ha^{-1}) were included in the network. Groups refer to the scope of differente experiments (G1 for event selection, G2 for genotype × environment evaluation and G3 for physiological analysis). Within each column, the color scale goes from dark green for the smallest value to dark red for the largest value, except for Rain+IR (opposite scale). ID: identification; SRc: cumulative incident solar radiation; Tmax: mean daily maximum temperature; Tmin: mean daily minimum temperature; Tmean: mean daily mean temperature; PET: potential evapotranspiration; Rain+IR: rainfall+irrigation; WB: water balance (WB= Rain+IR-PET)); RWB: relative water balance (RWB= $\frac{\text{Rain+IR}-\text{PET}}{\text{PET}}$).

Site in map	Harvest year	Experiment description	Water management	ID	Groups	SRc	Tmax	Tmin	Tmean	PET	Rain+IR	WB	RWB
						MJ m^{-2}	°C	°C	°C	mm	mm	mm	
1	2013	01. Aranguren ES	Rainfed	AR1a	G1, G2	2726	30.8	17.2	24.0	618	365	-253	-0.41
1	2013	02. Aranguren DS	Rainfed	AR1b	G1, G2	2263	30.2	16.3	23.1	641	310	-331	-0.52
1	2014	03. Aranguren MAP_0	Rainfed	AR2a	G2	1714	29.1	17.7	23.4	360	325	-35	-0.10
1	2014	04. Aranguren MAP_100	Rainfed	AR2b	G2	1714	29.1	17.7	23.4	360	325	-35	-0.10
2	2013	05. Carmen de Areco ES	Rainfed	CA1	G1, G2	2990	29.8	15.6	22.8	644	496	-148	-0.23
2	2013	06. Carmen de Areco DS	Rainfed	CA2	G1, G2	2915	27.5	13.9	20.6	615	379	-236	-0.38
3	2013	07. Corral de Bustos	Rainfed	CB	G1, G2	2765	29.0	15.1	21.8	574	337	-237	-0.41
4	2013	08. Chilibroste	Rainfed	CH	G1, G2	2908	30.6	15.7	22.8	646	407	-239	-0.37
5	2012	09. Hughes	Rainfed	HU1	G1, G2	2571	25.8	13.5	19.3	766	539	228	0.30
5	2013	10. Hughes	Rainfed	HU2	G1, G2	2668	28.0	14.8	21.3	575	341	-234	-0.41
6	2018	11. IAL-Santa Fe	Irrigated	IAL	G2, G3	2884	32.4	19.1	25.6	688	766	78	0.11
7	2013	12. Landeta	Rainfed	LA	G1, G2	2272	28.2	14.3	21.0	489	329	-160	-0.33
8	2010	13. Liborio Luna R	Rainfed	LL1	G2, G3	2586	29.0	14.6	21.4	564	404	-161	-0.28
8	2010	14. Liborio Luna IR	Irrigated	LL2	G2, G3	2586	29.0	14.6	21.4	564	570	5	0.01
9	2013	15. Monte Buey	Rainfed	MB1	G1, G2	3440	28.9	15.0	21.7	744	323	-421	-0.57
9	2014	16. Monte Buey	Rainfed	MB2	G2	2194	28.2	15.6	21.8	478	580	102	0.21
10	2018	17. Pergamino WD	Water Deficit	Pe1_D	G2, G3	2637	31.6	16.5	23.9	602	152	-451	-0.75
10	2018	18. Pergamino IR	Irrigated	Pe1_I	G2, G3	2637	31.6	16.5	23.9	602	332	-270	-0.45
10	2019	19. Pergamino IR	Irrigated	Pe2_I	G2, G3	2624	27.8	16.2	22.1	569	682	113	0.20
10	2019	20. Pergamino R	Rainfed	Pe2_R	G2, G3	2624	27.8	16.2	22.1	569	806	237	0.42
11	2010	21. Quimili	Rainfed	QU	G1, G2	1868	32.5	20.3	26.1	451	377	-75	-0.17
12	2014	22. Roldán ES	Rainfed	RO1	G2	2169	30.3	18.3	24.2	502	475	-27	-0.05
12	2014	23. Roldán DS	Rainfed	RO2	G2	2230	27.7	16.2	21.8	480	571	91	0.19
13	2013	24. San Agustín ES	Rainfed	SA1	G1, G2	3172	25.6	12.0	18.4	637	350	-287	-0.45
13	2013	25. San Agustín DS	Rainfed	SA2	G1, G2	3002	25.5	11.9	18.3	602	376	-226	-0.38
14	2013	26. Villa Saboya	Rainfed	VS1	G1, G2	2426	29.0	14.7	21.6	528	252	-276	-0.52
14	2014	27. Villa Saboya	Rainfed	VS2	G2	2306	28.9	15.7	22.2	508	459	-49	-0.10
